# A comparison of methods for analysing multiple outcome measures in randomised controlled trials using a simulation study

**DOI:** 10.1002/bimj.201900040

**Published:** 2020-12-14

**Authors:** Victoria Vickerstaff, Gareth Ambler, Rumana Z. Omar

**Affiliations:** ^1^ Division of Psychiatry University College London London UK; ^2^ Department of Statistical Science University College London London UK

**Keywords:** multiple endpoints, multiple outcomes, multivariate model, randomised controlled trials

## Abstract

Multiple primary outcomes are sometimes collected and analysed in randomised controlled trials (RCTs), and are used in favour of a single outcome. By collecting multiple primary outcomes, it is possible to fully evaluate the effect that an intervention has for a given disease process. A simple approach to analysing multiple outcomes is to consider each outcome separately, however, this approach does not account for any pairwise correlations between the outcomes. Any cases with missing values must be ignored, unless an additional imputation step is performed. Alternatively, multivariate methods that explicitly model the pairwise correlations between the outcomes may be more efficient when some of the outcomes have missing values. In this paper, we present an overview of relevant methods that can be used to analyse multiple outcome measures in RCTs, including methods based on multivariate multilevel (MM) models. We perform simulation studies to evaluate the bias in the estimates of the intervention effects and the power of detecting true intervention effects observed when using selected methods. Different simulation scenarios were constructed by varying the number of outcomes, the type of outcomes, the degree of correlations between the outcomes and the proportions and mechanisms of missing data. We compare multivariate methods to univariate methods with and without multiple imputation. When there are strong correlations between the outcome measures (*ρ* > .4), our simulation studies suggest that there are small power gains when using the MM model when compared to analysing the outcome measures separately. In contrast, when there are weak correlations (*ρ* < .4), the power is reduced when using univariate methods with multiple imputation when compared to analysing the outcome measures separately.

## 1 INTRODUCTION

In most clinical trials, a single primary outcome is specified to investigate the effect of a health intervention and this is often sufficient to determine whether the intervention is effective. However, for many diseases and disorders, a patient's health status cannot be adequately quantified using a single primary outcome. Examples include mental health disorders, stroke (Mayo & Scott, 2011) and chronic obstructive pulmonary disease (COPD) (Agusti & Vestbo, 2011; De Los Reyes, Kundey, & Wang, 2011; Teixeira‐Pinto, Siddique, Gibbons, & Normand, 2009). Therefore, in these disease areas, multiple primary outcomes may be required to provide a comprehensive understanding of the effects of an intervention.

These multiple outcomes may be of the same data type. For example, several continuous outcomes may be measured to quantify cognitive and behavioural components in order to evaluate the effect of cognitive behavioural therapy on patients with a depressive disorder. Alternatively, the outcomes may be of different data types. For example, researchers might measure a continuous quality of life outcome and a binary outcome to indicate symptom relapse when evaluating the effect of an antipsychotic drug on people with schizophrenia.

However, missing outcome data are a common problem for randomised controlled trials (RCTs) since it is not always possible to measure all specified primary outcomes for all participants. In fact, a review of published trials showed that outcome data were missing in the majority of trials (Bell, Fiero, Horton, & Hsu, 2014). Missing outcome data will generally result in a loss of power and may lead to biased estimates of the effect of the intervention. For example, patients in a smoking cessation trial may be more likely to drop out if they continue to smoke, and therefore the patients with observed outcome data may not be a representative sample.

Several approaches have been used to analyse trials with multiple outcomes in the presence of missing data. A common approach, which is appealing due its simplicity, has been to analyse the outcomes separately within a univariate framework (Vickerstaff, Ambler, King, Nazareth, & Omar, 2015). Patients are typically omitted from any analysis for which they have missing outcome data. However, this approach does not account for the correlation between the outcomes and consequently the precision of the estimates and the power may be lower than that achieved by other approaches (Teixeira‐Pinto et al., 2009). A variation on this approach is to use multiple imputation to impute missing outcome data prior to univariate analysis of the outcomes (White, Royston, & Wood, 2011). An advantage of this approach is that all outcomes may be included in the imputation model and hence the correlation between the outcomes may be accounted for (White et al., 2011).

More advanced approaches include the use of multivariate methods such as the multivariate multilevel (MM) model and multivariate regression. These methods have been used to analyse examination results in schools (Goldstein et al., 1993; Yang, Goldstein, Browne, & Woodhouse, 2002), crime trends (Mohan, Twigg, & Taylor, 2011; Tseloni & Zarafonitou, 2008) and health‐related behaviour (Maas, Verheij, Spreeuwenberg, & Groenewegen, 2008). However, the use of these methods in trials has been limited despite their potential to increase power (Snijders & Bosker, 2012). For example, the MM model has occasionally been used for exploratory analyses in clinical trials (Hassiotis et al., 2009; King et al., 2002).

Our recent review of published trials in neurology and psychiatry showed that multiple primary outcomes were commonly used but often inadequately analysed (Vickerstaff et al., 2015).

When analysing multiple outcomes in a trial, it is important to control the familywise error rate (FWER), which is the probability of obtaining at least one false positive result across the outcomes. A common approach to control the FWER is to adjust the *p*‐values produced by each statistical test (Dmitrienko & D'Agostino, 2013). Another important consideration for a trial with multiple primary outcomes is the definition of power since there are a number of ways that this may be defined. When the objective of the trial is to detect an effect for at least one of the specified outcomes, it is recommended to use the disjunctive power (Bretz, Hothorn, & Westfall, 2010; Dmitrienko, Tamhane, & Bretz, 2009) which is the probability of detecting at least one true intervention effect across the outcomes as statistically significant (Westfall, Tobias, & Wolfinger, 2011).

In this paper, we compare univariate and multivariate methods for the analysis of clinical trials with correlated multiple primary outcomes in the presence of missing data. The paper is structured as follows. First, we describe two motivating case studies. Then we present an overview of relevant methods to analyse trials with correlated multiple primary outcomes. Finally, we present the results of a simulation study to compare the bias, power and FWER achieved by some of these methods.

## 2 MOTIVATING EXAMPLES

In this section, we describe two clinical trials that will be used to illustrate the different approaches to analysis. The first trial has three continuous outcomes whereas the second has a mixture of continuous and binary outcomes.

### 2.1 Laser in glaucoma and ocular hypertension trial, LiGHT

LiGHT is a two‐arm, individually RCT (Gazzard et al., 2019) that recruited 718 patients with ocular hypertension or glaucoma taken from six centres across the United Kingdom. The primary outcome is EQ‐5D, which measures quality of life. In addition, there are two glaucoma‐specific quality of life outcomes: Glaucoma Quality of Life (GQL) and Glaucoma Utility Index (GUI). There are strong correlations between the baseline values of these three outcomes (EQ‐5D and GQL r = −0.51; EQ‐5D and GUI r = 0.51; GQL and GUI r = −0.72). At 24‐month follow‐up, 652 patients provided some outcome data although only 586 participants provided data on all three outcomes. In total, 652 (9% missing), 586 (18% missing) and 602 (16% missing) patients provided data for EQ‐5D, GQL and GUI, respectively.

### 2.2 Ten Top Tips (10TT) trial

The 10TT is a two‐arm, individually RCT (Beeken et al., 2012, 2017) that recruited 537 obese patients from 14 general practices across England. The aim of the trial was to investigate the effect of the 10TT intervention in primary care on obesity, where the intervention consisted of a leaflet called ‘*Ten Top Tips’* listing target behaviours alongside advice on repetition and context stability (Beeken et al., 2012). The primary outcome is weight change (kg) and two important secondary outcomes are change in waist circumference (cm) and blood glucose level (mmol/L). Weight change and waist circumferences are continuous outcomes whereas blood glucose level is binary after being categorised into ‘standard’ and ‘high’ groups. High blood glucose is defined as levels greater than 7.0 mmol/L (Organisation, 2018) with 18% of the trial participants categorised in this group. Correlation coefficients measured at baseline show that weight is strongly correlated with waist circumference (r=0.78). There is a weak/moderate correlation between weight and blood glucose level (r=0.28) and blood glucose level and waist circumference (r=0.36). At 3 months, 388 participants provided data on at least one outcome variable, with 383 (29% missing), 378 (30% missing) and 330 (39% missing) values provided for weight change, waist circumference and blood glucose level, respectively.

## 3 STATISTICAL METHODS FOR THE ANALYSIS OF MULTIPLE OUTCOMES

In this section, we briefly describe relevant methods that have been proposed to analyse multiple correlated outcomes in clinical trials. Let us consider a two‐arm trial with n trial participants and m correlated primary outcomes. The ith trial participant is randomly assigned to either the intervention group $\left(\;x_{i}=\;1\right)$ or the control group $\left(\;x_{i}=\;0\right)$, for i=1,…,n. Let $\;Y_{ij}\;$ be the value of the jth outcome for the ith participant and $\;\beta_{1j}$ represents the effect of the intervention on the jth outcome. The aim of the trial is to test the null hypotheses $H_{0j}:\;\beta_{1j}=\;0$ for j=1,…,m, where the jth null hypothesis states that the intervention has no effect on the jth outcome. A test statistic $t_{j}$ is used to test each null hypothesis $H_{0j}$ and $p_{j}$ is the corresponding unadjusted *p*‐value. Further suppose that there is an overall null hypothesis $H\;\left(m\right)=\cap_{j=1}^{m}H_{j}$ and that the joint test statistic $(t_{1},\ldots ,\;t_{m})\;$has an *m*‐variate distribution. Statistical significance is set to α. For clarity, the subscript i for participants is omitted in most of the following notation and the models include no covariates in addition to that for the intervention. Unless otherwise stated, we consider only disjunctive power and hence an intervention is shown to be effective if a statistically significant result is found for at least one outcome.

### 3.1 Combined outcome

One straightforward approach to analysing multiple outcomes is to combine the outcomes into a single composite outcome. For example, to combine two time‐to‐event outcomes, we might consider the time until the first event (Dmitrienko et al., 2009). An example of this might be time from randomisation until either nonfatal ischemic stroke, fatal ischemic stroke or early death. The composite outcome approach avoids testing outcomes separately and the need to adjust *p*‐values (Phillips & Haudiquet, 2003). The composite outcome needs to be specified before the trial starts and all of its components should be of equal importance when assessing the effect of the intervention (Montori et al., 2005). A composite outcome may not be appropriate when the effects of an intervention differ in magnitude and/or direction across the outcomes (Pogue, Devereaux, Thabane, & Yusuf, 2012). In particular, the latter may result in a large loss of power.

### 3.2 Analysing outcomes separately

As discussed earlier, a common approach to analysing multiple outcomes is to analyse each outcome separately within a univariate framework. However, any correlations between the outcomes are not used and an extra imputation step may be required in the presence of missing data.

### 3.3 Multivariate analysis

More advanced techniques, including multivariate methods (Goldstein, 2011), have been proposed that enable multiples outcomes to be analysed simultaneously taking into account the correlations between them (Teixeira‐Pinto & Normand, 2009). The use of these methods could potentially lead to improved precision and greater power (McCulloch, 2008) and hence smaller sample sizes. In addition, depending on the objective of the trial, we may also estimate an overall effect of the intervention across outcomes, as well as a separate effect for each outcome.

#### 3.3.1 Global statistical tests

Another multivariate approach is to use a global testing procedure to estimate an overall effect of the intervention across outcomes, with the trial deemed a success if the overall effect is statistically significant. Conceptually, the interpretation of results obtained from global test procedures and the analysis of composite outcomes are similar, and both avoid the issues associated with testing outcomes separately. However, unlike composite outcomes, global test procedures account for the correlations between outcomes. Methods include the multivariate analysis of variance (MANOVA), the one‐degree of freedom global test developed by Roy (Roy, Lin, & Ryan, 2003) and the test statistics developed by O'Brien (O'Brien, 1984) and extended by Pocock et al. (Pocock, 1997). Global testing procedures require balanced data across all outcomes and will omit observations if any outcome values are missing. Given this limitation, global testing procedures are not widely used in clinical trials and therefore are not discussed further.

#### 3.3.2 Multivariate regression

Multivariate regression is an extension of multiple regression that allows for multiple outcomes of the same type to be analysed. For example, this approach may be used to analyse several continuous or several binary outcomes. Multivariate regression also requires balanced data across the outcomes.

#### 3.3.3 Factorisation modelling

This approach involves factorising the joint distribution of two correlated outcomes into a marginal distribution and a conditional distribution. Univariate (UV) models can then be fitted to both components of this factorisation (Teixeira‐Pinto & Harezlak, 2013). It is possible to use different types of outcomes within this framework although the estimated intervention effects are likely to be different from those obtained by analysing the outcomes separately because of different distributional assumptions.

With two correlated outcomes, one continuous ($Y_{1})$ and one binary ($Y_{2})$, we can use one of the two possible factorisations of their joint distribution $f_{Y_{1},\;Y_{2}}\;\left(y_{1},\;y_{2}\right)=f_{Y_{1}\;|\;Y_{2}\;}\;\left(y_{1}|y_{2}\right)f_{Y_{2}}\left(y_{2}\right)$. Fitzmaurice and Laird (1995) describe a factorisation model which uses a linear model for $Y_{1}$ and a probit model for $Y_{2}$, and includes a covariate for intervention group. The model is

(1) $$\begin{array}{c} Y_{1}=\;\beta_{01}+\;\beta_{11}x+\tau \;\left(Y_{2}-\mu_{2}\right)+\;\in_{1},\\ probit\;\left(\mu_{2}\right)=\beta_{02}+\;\beta_{12}\;x, \end{array}$$

where $\in_{1}∼N\left(0,\sigma_{c}^{2}\right)$ is a normally distributed random variable with mean zero and variance $\sigma_{c}^{2}$, and τ quantifies the association between $Y_{1}\;$and $Y_{2}$. Catalano and Ryan (1992) propose the ‘reverse’ of this model which uses the other possible factorisation $f_{Y_{1},\;Y_{2}}\;\left(y_{1},\;y_{2}\right)=f_{Y_{1}}\;\left(y_{1}\right)f_{Y_{2}\;|\;Y_{1}\;}\left(y_{2}|y_{1}\right).$ This is described in Teixeira‐Pinto and Harezlak (2013). At present, there is no guidance on how to analyse more than two outcomes using the factorisation model.

#### 3.3.4 Latent modelling

Several researchers have suggested methods that use latent variables (LVs) to model multiple correlated outcomes, including McCulloch (2008), Sammel, Ryan, and Legler (1997) and Dunson (2000). McCulloch (2008) suggests specifying a random effect l that is shared across outcomes. Assuming we have one continuous normally distributed outcome ($Y_{1})\;$and a binary outcome ($Y_{2})$, the model is

(2) $$\begin{array}{c} Y_{1}=\beta_{01}+\beta_{11}x+l+\in_{1}\\ P\left(Y_{2}=1\right)=\phi \left(\beta_{02}+\beta_{12}x+\lambda l\right), \end{array}$$

where $e_{1}∼\;N\left(0,\;\sigma_{1}^{2}\right)$, $l∼\;N(0,\;\sigma_{l}^{2})$ and $\sigma_{1}^{2}$ and $\sigma_{l}^{2}$ are unknown variances. We assume that the LV l completely specifies the pairwise correlation between the outcomes and hence, conditional on this variable, the two outcomes are independent. The parameter λ accounts for the fact that the linear predictors for $Y_{1}$ and $Y_{2}$ are on different scales and will have different variances.

The estimated effects of the intervention from this model are conditional on the LV and consequently they may not be comparable to the estimates obtained from the other methods discussed. To obtain estimates for binary outcomes that are comparable to those obtained from univariate analysis, we divide the regression coefficient $\beta_{12}$ by $\sqrt{\lambda^{2}\sigma_{l}^{2}+\;\sigma_{2}^{2}\;}$ (Teixeira‐Pinto & Normand, 2009), where $\sigma_{2}^{2}$ is fixed to 1 if a probit link function is used. A detailed discussion regarding coefficient adjustments can be found in Teixeira‐Pinto and Normand (2008). Note that in model (2), there are three variance–covariance parameters ($\sigma_{1}^{2},\;\sigma_{l}^{2}$ and λ) that need to be estimated. However, there are only two quantities we can use to estimate these parameters and hence, it is necessary to impose an additional constraint to ensure that the model is not over parameterised and the model parameters are identifiable (Teixeira‐Pinto & Normand, 2009). One option is to fix the variance of the LV $\sigma_{l}^{2}$. A similar restriction is needed when analysing multiple continuous or multiple binary outcomes. McCulloch (2008) also investigated the use of this model for other types of outcomes. Sammel et al. (1997) discuss another LV model for mixed discrete and continuous outcomes which allows the use of any distribution from the exponential family.

#### 3.3.5 The MM model

The MM model has been suggested as another approach to analyse correlated multiple outcomes. In the MM model, multiple outcomes are considered to be nested within individuals and are treated in a similar manner to how repeated measurements are treated within the multilevel modelling framework (Goldstein, 2011, Goldstein, Carpenter, Kenward, & Levin, 2009). For two continuous outcomes $Y_{1}$ and $Y_{2}$, the following model is used:

(3) $$\begin{array}{c} \;Y_{j}=z_{1j}\;\left(\beta_{01}+\beta_{11}x+\in_{1\;}\right)+z_{2j}\left(\beta_{02}+\beta_{12}x+\in_{2\;}\right),\\ z_{1j}=\;1\;\mathrm{if}j\;=\;1\;\mathrm{and}\;z_{1j}=\;0\;\mathrm{otherwise},\\ z_{2j}=\;1-z_{1j}, \end{array}$$

where $z_{kj}$ is an indicator for outcome $Y_{j}$ and $\in_{j}∼N\left(0,\Omega_{u}\right)$is the random error for the level two structure where $\Omega_{u}$ is the unknown covariance matrix. Level one variation is not specified as the level exists solely to define the multivariate structure. The formulation as a multilevel model allows for estimation of a covariance matrix even if some of the outcome data are missing, as long as missingness at random (Goldstein, 2011). In the above model, two intervention effects are specified, one for each outcome. However, a common effect across both outcomes can also be specified. In addition, the model can handle mixed outcome types (Goldstein et al., 2009).

#### 3.3.6 Summary of the multivariate methods

The factorisation, LV and MM models can handle continuous outcomes, binary outcomes or a mixture of both. In addition, these models can handle nonoverlapping missingness, where values may be missing for some but not all of some of the outcomes. That is, the number of observations does not need to be balanced across outcomes. Also, the factorisation, LV and MM models can easily be extended to handle several outcomes although the factorisation model can be cumbersome with more than two outcomes.

## 4 SIMULATION STUDY

In this section, we use simulation to compare the MM and LV models to univariate models with (MI+UV) and without multiple imputation (UV) with respect to power and FWER. Recommendations are made regarding which of these methods provide the most power while controlling the FWER. Several scenarios were considered by varying the number of outcomes, the outcome type, the correlation between outcomes, the size of the intervention effect, the missing data mechanism and the percentage of missing data values. Details of the different simulation factors are described in Table 1.

**TABLE 1:** The simulation factors

Variable	Simulation factors
Number of outcomes	2 or 4
Outcome type	Continuous; binary; mixed (half‐continuous and half‐binary)
Correlation between outcomes	.0,.2,.4,.6,.8
Effect size (ES) of intervention effect	*Continuous outcomes* Equal: ES = 0.35 for all outcomes. Varying: $\mathrm{ES}\;={\left(0.2,\;0.4\right)}^{T}\;$ or $\mathrm{ES}\;={\left(0.1,\;0.2,\;0.3,\;0.4\right)}^{T}\;$ for two and four outcomes, respectively. *Binary outcomes* Equal: 50% and 65% event rate in control and intervention arms, respectively, for all outcomes. *Mixed outcomes* Constant: ES = 0.35 for all outcomes.
Missing data mechanism	Missing completely at random (MCAR), missing at random (MAR), missing not at random (MNAR)
Percentage of missing data values	Low and high levels of missingness. Percentages varied on depending on the missingness mechanisms and the number of outcomes, as described below: *MCAR and MAR, two outcomes* Low: 15% and 25% missing values in outcomes 1 and 2 High: 30% and 50% missing values in outcomes 1 and 2 *MCAR and MAR, four outcomes* Low: 15%, 15%, 25% and 25% missing values in outcomes 1, 2, 3 and 4 High: 20%, 30%, 40% and 50% missing values in outcomes 1, 2, 3 and 4 *MNAR, two outcomes* Low: 15% missing values in outcome 1 High: 50% missing values in outcome 1 High overlapping: 30% and 50% of observations in outcomes 1 and 2 were missing. *MNAR, four outcomes* Low: 15% missing values in outcomes 1 and 2 High: 50% missing values in outcomes 1 and 2 High overlapping: 20%, 30%, 40% and 50% of observations were missing for each of the outcomes respectively.

We used the following model to simulate outcome values for two continuous outcomes $Y_{i}=\;(Y_{i,1},\;Y_{i,2}$),

$$Y_{i}=\;\beta_{0}+\;\beta_{1}x_{i}+\;\varepsilon_{i},$$

where $x_{i}$ indicates whether participant i received intervention ($x_{i}=\;1$) or control ($x_{i}=\;0$), $\beta_{1}={\left(\;\beta_{11},\;\beta_{12}\;\right)}^{T}\;$ is a vector of intervention effects for each outcome, $\in_{i}$ are errors which are realisations of a multivariate normal distribution

$$\varepsilon_{i}={\left(\varepsilon_{i,1},\varepsilon_{i,2}\;\right)}^{T}∼N\left(\;\left(\begin{array}{c} 0\\ 0 \end{array}\right),\;\left(\begin{array}{cc} 1 & \rho \\ \rho & 1 \end{array}\right)\;\right),$$

where ρis the correlation between outcomes. This model was extended in an obvious way to simulate four continuous outcomes. A similar approach was used to simulate binary outcomes, with an extra final step to dichotomise the continuous outcomes at zero.

The sample size was set at 260 for the continuous and mixed scenarios and 340 for the binary scenarios, with equal numbers of participants being allocated to the two intervention groups. These numbers were obtained from sample size calculations for a single outcome using the equal effect sizes in Table 1, 5% statistical significance and 80% power.

We introduced missing data under a variety of assumptions. We use three forms of missingness: (a) missingness completely at random (MCAR), (b) missing at random (MAR) and (c) missing not at random (MNAR). Missingness was implemented by simulating values from a multivariate Bernoulli distribution (Leisch et al., 1998) and setting the outcome variables to be missing according to the corresponding binary indicator. In the MAR scenarios, the probability of missingness depends on the intervention group with outcomes being 1.5 times more likely to be missing in the control arm compared to the intervention arm. To simulate the data under an MNAR mechanism, the probability of missingness was set to increase with increasing outcome values. First, a data set with no missing values was simulated. Then the observations were sorted in ascending order based on the outcome in which missing values were to be introduced and divided into quartile groups. Missingness was then introduced randomly into each quartile group using Table 2.

**TABLE 2:** The percentage of missing observations per quartile used to simulate MNAR data

	Percentage of observations missing per quartile			
Percentage of missing observations	First	Second	Third	Fourth
0%	0	0	0	0
15%	0	7.5	22.5	30
20%	0	10	30	40
30%	0	15	45	60
40%	0	20	60	80
50%	0	25	75	100

To estimate the FWER, we specified that the intervention had no effect ($\beta_{1}=\;0$) then calculated the proportion of times that a significant test result was observed for at least one of the outcomes over 10,000 simulations. The Holm adjustment was used to control the FWER (Holm, 1979). To estimate the disjunctive power, a similar approach was used with a specified intervention effect ($\beta_{1}\;\neq 0)$. The bias in the estimated effects was calculated as the difference between the average effect estimates over simulations and the true values. Monte Carlo standard errors (MCSE) were also calculated to provide an estimate of simulation accuracy for each scenario.

The following methods of analysis were used:

1. UV models. This was used as the comparator for the other methods.
2. MI+UV models.
3. MM model.
4. LV model.

For the univariate approach, continuous outcomes were analysed using a linear regression model and binary outcomes were analysed using a probit regression model. The latter was used as it corresponds to how the data were generated.

Multiple imputation was implemented using chained equations (MICE) since this is one of the most widely used methods to impute missing data (Sterne et al., 2009). Outcomes in the two arms were imputed separately which is equivalent to imputing missing values conditional on intervention arm. Forty imputations were used for all scenarios, which is the recommended number of imputations when 50% of the data are missing (Graham, Olchowski, & Gilreath, 2007). Estimates were pooled across imputed data sets using Rubin's rules (Rubin, 2004). The LV models used adaptive quadrature (Rabe‐Hesketh, Skrondal, & Pickles, 2005) with 10 integration points to fit the models by maximum likelihood estimation. To ensure that the model is not over parameterised, we fixed the latent factor variance to 0.8 (Grilli & Rampichini, 2006) in the scenarios with continuous and mixed outcomes. In the scenarios with binary outcomes, we fixed the latent factor to 1. The MM model was implemented in MLwiN via R using the package ‘R2MLwiN’. The MI+UV model was implemented using the ‘mice’package in R; and the LV method was implemented using GLLAMM (www.gllamm.org) in Stata Release 14 (StataCorp, 2015).

### 4.1 Results

#### 4.1.1 MCAR and MAR mechanisms

The results for the different methods in different simulation scenarios with either MCAR or MAR missing data mechanisms are shown in Tables 3a, 3b and 3c. When analysing two binary outcomes the MM model occasionally did not converge, most frequently when the correlation between the outcomes was strong (ρ=.8) and there was no effect of intervention. When analysing four binary outcomes, the MM model often did not converge and consequently we do not report these results. The MCSE for the estimates of FWER and disjunctive power were similar for all methods. The MCSE ranged from 0.0016 to 0.0026 for the FWER estimates and from 0.0026 to 0.0050 for the disjunctive power estimates.

##### Bias

The effect estimates were unbiased for the UV and MI+UV approaches when analysing continuous outcomes (results shown in the Appendix) since the intervention group, which was the predictor of missingness, was included in the models. However, a small bias was observed when analysing two binary outcomes using the MI+UV method (results shown in the Appendix). This may be because the MICE routine in R requires us to use logistic regression to impute missing values for binary variables instead of probit regression, which was used to simulate the binary outcomes. The effect estimates were unbiased when analysing two continuous outcomes using the MM model (results shown in the Appendix).

##### FWER

The FWER was around the 5% level for the UV approach for all outcome types, whereas it varied between 2.8% and 5.5% for the MI+UV approach when analysing continuous outcomes and was slightly conservative when analysing binary outcomes. The FWER fluctuated around the 5% level for the MM model, with the highest level of 5.8% observed when there were high levels of missing data. When using the LV model, the FWER ranged from 2.7% to 5.7% when analysing continuous and mixed outcomes.

##### Disjunctive power

The MM and LV models show increased disjunctive power compared to the UV and MI+UV approaches when analysing two continuous outcomes with missing data. These models show power gains over the UV approach even when there is weak correlation, although these gains are modest when the proportion of missing data is less than 30%. The MI+UV approach had lower disjunctive power than the UV approach with two continuous outcomes in weak to moderate correlation scenarios. When there is zero or weak correlation (ρ=.2) between the outcomes, the imputed outcome values are highly variable which leads to slightly higher empirical standard errors for the effect estimates compared to those obtained by the UV approach (shown in the Appendix). Consequently, disjunctive power is reduced when using the MI+UV approach, particularly when the correlations between outcomes are weak to moderate. When the outcomes are strongly correlated and missing data are not overlapping across outcomes, the observed outcome values are highly predictive of the missing outcome values which lead to smaller empirical standard errors for the MI+UV approach and disjunctive power similar to that of the MM and LV models. When analysing four continuous outcomes, the performance of the MI+UV approach is slightly improved (results presented in the Appendix), although the disjunctive power is still slightly lower than that of the UV approach when the outcomes are uncorrelated.

With two binary outcomes, the disjunctive power of the MM model but was slightly higher than that of the other approaches. With mixed outcomes, the MI+UV approach and the MM model performed similarly with slightly higher disjunctive power than that of the UV approach. The LV model had the lowest power when the outcome correlation was 0.6 or higher.

Similar results are observed when analysing four continuous outcomes or when there are varying effect sizes (results presented in the Appendix). The MM model was the only multivariate approach we considered in these scenarios, and the MNAR scenario, as its performance was superior to that of the LV model in the previous simulations.

##### MNAR mechanism

**FIGURE 1:**
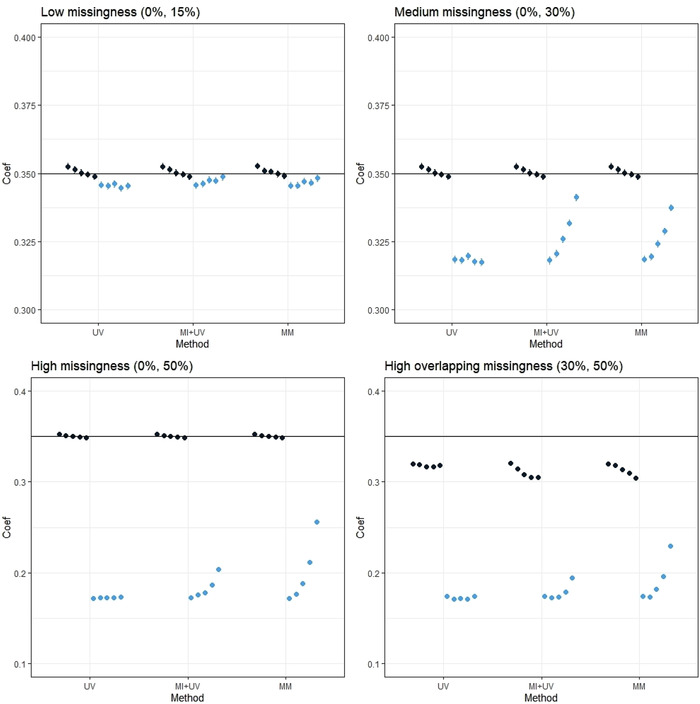
Bias in estimating the intervention effects when simulating two continuous outcomes and data are MNAR *Note*. The dark blue dots represent the average of the estimated treatment effects ($\hat{\beta }$) for outcome 1. The light blue dots represent the average of the estimated treatment effects ($\hat{\beta }$) for outcome 2. The five dots (of either colour) clustered together represent different correlations between outcomes from 0 (left) to .8 (right) in increments of .2. Each graph corresponds to a different level of missing data as indicated. The true intervention effect is represented by the black horizontal line. The Monte Carlo standard errors for the estimated bias was between .0005 and .0007 for all scenarios

Both the MM model and MI+UV approach assume that the missing values are MAR. We generated missing values under an MNAR mechanism to investigate if bias in the effect estimates could be reduced by using the correlation between outcomes. As expected, use of either the MM model or MI+UV approach did not reduce bias when the outcomes were uncorrelated. However, bias was reduced when the outcomes were strongly correlated and there were high levels of missing data. In particular, there was a notable reduction in bias when the outcome correlation exceeded 0.4. However, neither approach was able to remove the bias entirely. Results are shown in Figure 1.

## 5 CASE STUDIES

### 5.1 Reanalysis of the LiGHT and 10TT trials

We now reanalyse the two real data sets, LiGHT and 10TT, to illustrate differences and similarities between the MM, MI+UV, LV and UV approaches. The code used to implement the MM model in Stata, R and MlwiN is provided in the Appendix.

#### 5.1.1 Laser in glaucoma and ocular hypertension trial, LiGHT

In this analysis, the 24‐month outcomes were used and the corresponding baseline values for each outcome were adjusted for in the models.

On the EQ‐5D and GUI scales, a higher score means better quality of life, whereas on the GQL scale, a higher score means poorer quality of life. When using the MM model, the GQL scale was reversed to enable the estimation of intervention effects that are in the same direction.

The results for the four models are displayed in Table 4. The UV approach uses a different number of participants for each outcome depending on the amount of missingness whereas the MI+UV, MM and LV approaches use all 652 participants for the analyses. The standard errors of the effect estimates are very similar across the approaches.

**TABLE 4:** Analysis of the LiGHT data set using the UV, MI+UV, MM and LV models

	*N*	Mean diff.^a^	SE^a^	95% CI^a^	*p*‐Value
*UV*					
EQ‐5D	652	−0.000	0.008	(−0.016, 0.015)	.967
GQL	586	−0.476	0.457	(−1.373, 0.422)	.298
GUI	602	0.014	0.008	(−0.001, 0.029)	.063
*MI+UV*					
EQ‐5D	652	−0.000	0.008	(−0.016, 0.015)	.958
GQL	652	−0.559	0.449	(−1.44, 0.323)	.213
GUI	652	0.015	0.008	(0.000, 0.030)	.052
*MM*					
EQ‐5D	652	−0.000	0.008	(−0.016,0.015)	.954
GQL	652	−0.455	0.454	(−1.344,0.435)	.317
GUI	652	0.014	0.008	(−0.001,0.029)	.071
*LV*					
EQ‐5D	652	−0.001	0.008	(−0.016, 0.014)	.873
GQL	652	−0.392	0.425	(−1.23, 0.442)	.357
GUI	652	0.013	0.007	(−0.001, 0.026)	.072

^a^ Standardised intervention effects.

Abbreviations: CI, confidence interval; GQL, glaucoma quality of life scale; GUI, glaucoma utility index; LV, latent variable model; Mean diff, mean difference; MM, multilevel multivariate model; SE, standard error; UV, univariate model; MI+UV, multiple imputation followed by univariate model.

In summary, similar results are obtained from all approaches possibly due to the relatively small proportion of missing data. One advantage of the MM model is that a joint effect could also be calculated, if appropriate, for some or all of the outcomes. For example, a joint effect could be estimated for the glaucoma specific scales GUI and GQL while simultaneously estimating an individual effect for EQ‐5D. In a trial scenario, the decision to estimate a joint effect would need to be made at the start of the study and documented in the statistical analysis plan.

#### 5.1.2 The 10TT trial

For the analysis of the 10TT trial, the outcomes were standardised and the corresponding baseline variables were adjusted for in the various models.

The estimated effect for blood glucose differs slightly between the models (Table 5) which may be due to higher proportion of missing data for this outcome. In summary, the MM and LV models allow both continuous and binary outcomes to be analysed simultaneously. However, we found that in this trial reanalysis, use of the MM and LV models made little difference to the results and conclusions when compared to those obtained using the UV approach.

**TABLE 5:** Analysis of Ten Top Tip data set using UV, MI+UV, MM and LV models

	*N*	Coef.	Standard error	95% Confidence interval	*p*‐Value
*UV*					
Weight change	383	−0.052	0.018	(−0.088, −0.016)	.004
Waist circumference	378	−0.069	0.0483	(−0.164, 0.026)	.153
Blood glucose^a^	330	−0.151	0.298	(−0.734, 0.433)	.612
*MI+UV*					
Weight change	388	−0.052	0.018	(−0.088, −0.016)	.004
Waist circumference	388	−0.074	0.048	(−0.169, 0.020)	.122
Blood glucose^a^	388	−0.174	0.304	(−0.770, 0.421)	.566
*MM*					
Weight change	388	−0.053	0.018	(−0.088, −0.016)	.004
Waist circumference	388	−0.071	0.048	(−0.165, 0.023)	.141
Blood glucose^a^	388	−0.180	0.295	(−0.759, 0.398)	.542
*LV*					
Weight change	388	−0.054	0.024	(−0.102, −0.008)	.021
Waist circumference	388	−0.092	0.123	(−0.333, −0.150)	.455
Blood glucose^a^	388	−0.194	0.214	(−0.614, 0.225)	.364

^a^ Blood glucose as a categorical variable: normal/high.

Abbreviations: LV, latent variable model; MM, multilevel multivariate model; UV, univariate model; MI+UV, multiple imputation followed by univariate model.

**TABLE 3a:** FWER and disjunctive power when analysing two continuous outcomes

		*ρ* ↓	Familywise error rate (FWER)				Disjunctive power				Relative power (vs. UV)		
Type of Missingness ↓	% of missing values for each outcome ↓	Method →	UV	MI+UV	MM	LV	UV	MI+UV	MM	LV	MI+UV	MM	LV
Complete	(0%, 0%)	0	0.052	‐	0.054	0.054	0.922	‐	0.926	0.926	‐	1	1
		.2	0.048	‐	0.051	0.051	0.894	‐	0.9	0.9	‐	1.01	1.01
		.4	0.052	‐	0.054	0.054	0.873	‐	0.877	0.877	‐	1	1
		.6	0.045	‐	0.048	0.048	0.838	‐	0.844	0.844	‐	1.01	1.01
		.8	0.038	‐	0.04	0.048	0.798	‐	0.806	0.814	‐	1.01	1.02
MCAR	(15%, 25%)	0	0.048	0.044	0.051	0.05	0.852	0.811	0.86	0.859	0.95	1.01	1.01
		.2	0.049	0.045	0.054	0.053	0.825	0.804	0.835	0.835	0.97	1.01	1.01
		.4	0.048	0.051	0.053	0.053	0.803	0.807	0.818	0.818	1	1.02	1.02
		.6	0.048	0.051	0.05	0.049	0.769	0.79	0.797	0.797	1.03	1.04	1.04
		.8	0.044	0.046	0.044	0.052	0.741	0.779	0.779	0.787	1.05	1.05	1.06
MCAR	(30%, 50%)	0	0.05	0.028	0.055	0.055	0.721	0.532	0.738	0.736	0.74	1.02	1.02
		.2	0.05	0.029	0.058	0.057	0.704	0.568	0.726	0.725	0.81	1.03	1.03
		.4	0.051	0.044	0.056	0.055	0.689	0.648	0.718	0.716	0.94	1.04	1.04
		.6	0.049	0.055	0.054	0.055	0.667	0.694	0.711	0.711	1.04	1.07	1.07
		.8	0.044	0.052	0.045	0.053	0.643	0.708	0.704	0.709	1.1	1.09	1.1
MAR	(15%, 25%)	0	0.052	0.048	0.055	0.055	0.849	0.808	0.858	0.858	0.95	1.01	1.01
		.2	0.049	0.045	0.053	0.052	0.825	0.805	0.834	0.834	0.98	1.01	1.01
		.4	0.05	0.051	0.053	0.053	0.794	0.8	0.809	0.809	1.01	1.02	1.02
		.6	0.047	0.051	0.052	0.052	0.765	0.784	0.788	0.788	1.02	1.03	1.03
		.8	0.044	0.046	0.046	0.054	0.726	0.761	0.761	0.769	1.05	1.05	1.06
MAR	(30%, 50%)	0	0.05	0.032	0.056	0.056	0.713	0.539	0.729	0.728	0.76	1.02	1.02
		.2	0.048	0.032	0.052	0.052	0.696	0.567	0.718	0.716	0.81	1.03	1.03
		.4	0.048	0.042	0.053	0.052	0.678	0.631	0.702	0.701	0.93	1.04	1.03
		.6	0.05	0.054	0.053	0.053	0.656	0.67	0.696	0.696	1.02	1.06	1.06
		.8	0.04	0.052	0.047	0.055	0.627	0.678	0.677	0.685	1.08	1.08	1.09

*Note*. Multivariate and univariate methods are compared for scenarios which vary in terms of missingness, percentage of missing data and degree of correlation between outcomes. The Holm method was applied to all scenarios to account for multiplicity. Monte Carlo standard errors (MCSE) were consistent across methods. MCSE Range for FWER = (0.0017, 0.0023); MCSE Range for Power = (0.0026, 0.0050).

Abbreviations: LV, latent variable model; MM, multilevel multivariate model; UV, univariate model; MI+UV, multiple imputation followed by univariate model; *ρ* , correlation between outcomes.

**TABLE 3b:** FWER and disjunctive power when analysing two binary outcomes

		*ρ* ↓	Familywise error rate (FWER)				Disjunctive power				Relative power (vs. UV)		
Type of missingness ↓	% of missing values for each outcome ↓	Method →	UV	MI+UV	MM	LV	UV	MI+UV	MM	LV	MI+UV	MM	LV
Complete	(0%, 0%)	0	0.053	‐	0.064	0.053	0.919	‐	0.92	0.919	‐	1.00	1.00
		.2	0.047	‐	0.052	0.047	0.902	‐	0.903	0.902	‐	1.00	1.00
		.4	0.049	‐	0.058	0.049	0.893	‐	0.895	0.893	‐	1.00	1.00
		.6	0.053	‐	0.064	0.052	0.863	‐	0.865	0.861	‐	1.00	1.00
		.8	0.047	‐	0.058	0.036	0.84	‐	0.843	0.821	‐	1.00	0.98
MCAR	(15%, 25%)	0	0.051	0.049	0.07	0.05	0.848	0.838	0.847	0.844	0.99	1.00	1.00
		.2	0.048	0.044	0.063	0.044	0.836	0.824	0.838	0.828	0.99	1.00	0.99
		.4	0.044	0.043	0.061	0.042	0.816	0.811	0.824	0.81	0.99	1.01	0.99
		.6	0.05	0.049	0.068	0.046	0.795	0.792	0.805	0.786	1.00	1.01	0.99
		.8	0.046	0.043	0.063	0.036	0.768	0.776	0.792	0.75	1.01	1.03	0.98
MCAR	(30%, 50%)	0	0.051	0.045	0.066	0.048	0.729	0.704	0.734	0.721	0.97	1.01	0.99
		.2	0.048	0.042	0.058	0.043	0.715	0.693	0.72	0.698	0.97	1.01	0.98
		.4	0.047	0.043	0.062	0.042	0.701	0.681	0.71	0.678	0.97	1.01	0.97
		.6	0.052	0.046	0.063	0.041	0.687	0.671	0.707	0.656	0.98	1.03	0.95
		.8	0.048	0.041	0.063	0.034	0.667	0.672	0.708	0.627	1.01	1.06	0.94
MAR	(15%, 25%)	0	0.049	0.046	0.068	0.048	0.849	0.84	0.85	0.846	0.99	1.00	1.00
		.2	0.05	0.046	0.067	0.048	0.832	0.824	0.835	0.827	0.99	1.00	0.99
		.4	0.046	0.042	0.06	0.042	0.812	0.806	0.819	0.808	0.99	1.01	1.00
		.6	0.048	0.045	0.063	0.044	0.792	0.789	0.801	0.783	1.00	1.01	0.99
		.8	0.049	0.044	0.064	0.036	0.767	0.769	0.786	0.745	1.00	1.02	0.97
MAR	(30%, 50%)	0	0.052	0.048	0.069	0.05	0.722	0.700	0.726	0.715	0.97	1.01	0.99
		.2	0.049	0.043	0.058	0.044	0.707	0.686	0.709	0.691	0.97	1.00	0.98
		.4	0.048	0.042	0.064	0.042	0.686	0.673	0.697	0.67	0.98	1.02	0.98
		.6	0.051	0.044	0.065	0.041	0.672	0.665	0.689	0.649	0.99	1.03	0.97
		.8	0.048	0.041	0.065	0.034	0.657	0.661	0.687	0.616	1.01	1.05	0.94

*Note*. Multivariate and univariate methods are compared for scenarios which vary in terms of missingness, percentage of missing data and degree of correlation between outcomes. The Holm method was applied to all scenarios to account for multiplicity. Monte Carlo standard errors for were consistent across methods. MCSE Range for FWER = (0.0018, 0.0026); MCSE Range for Power = (0.0027, 0.0049).

Abbreviations: LV, latent variable model; MM, multilevel multivariate model; UV, univariate model; MI+UV, multiple imputation followed by univariate model; ρ, correlation between outcomes.

**TABLE 3c:** FWER and disjunctive power when analysing one continuous and one binary outcome (mixed outcomes)

		*ρ* ↓	Familywise error rate (FWER)				Disjunctive power				relative power (vs. UV)		
Type of missingness ↓	% of missing values for each outcome ↓	Method →	UV	MI+UV	MM	LV	UV	MI+UV	MM	LV	MI+UV	MM	LV
Complete	(0%, 0%)	0	0.052	‐	0.054	0.054	0.861	‐	0.864	0.864	‐	1	1
		.2	0.053	‐	0.054	0.054	0.836	‐	0.84	0.84	‐	1	1
		.4	0.051	‐	0.052	0.052	0.815	‐	0.819	0.816	‐	1	1
		.6	0.046	‐	0.048	0.04	0.794	‐	0.798	0.779	‐	1.01	0.98
		.8	0.045	‐	0.046	0.027	0.765	‐	0.77	0.723	‐	1.01	0.95
MCAR	(15%, 25%)	0	0.05	0.049	0.051	0.051	0.777	0.776	0.784	0.784	1	1.01	1.01
		.2	0.05	0.049	0.052	0.051	0.756	0.757	0.765	0.763	1	1.01	1.01
		.4	0.048	0.048	0.05	0.049	0.74	0.746	0.75	0.746	1.01	1.01	1.01
		.6	0.05	0.047	0.05	0.039	0.72	0.734	0.738	0.71	1.02	1.03	0.99
		.8	0.047	0.045	0.047	0.029	0.698	0.715	0.722	0.68	1.02	1.03	0.97
MCAR	(30%, 50%)	0	0.049	0.05	0.051	0.051	0.655	0.668	0.665	0.663	1.02	1.02	1.01
		.2	0.048	0.05	0.05	0.049	0.648	0.658	0.66	0.656	1.02	1.02	1.01
		.4	0.049	0.054	0.053	0.048	0.632	0.653	0.651	0.64	1.03	1.03	1.01
		.6	0.05	0.052	0.053	0.04	0.623	0.656	0.653	0.612	1.05	1.05	0.98
		.8	0.046	0.046	0.05	0.029	0.601	0.638	0.638	0.589	1.06	1.06	0.98
MAR	(15%, 25%)	0	0.05	0.049	0.051	0.051	0.782	0.779	0.786	0.786	1	1.01	1.01
		.2	0.048	0.047	0.051	0.051	0.757	0.755	0.764	0.763	1	1.01	1.01
		.4	0.052	0.049	0.052	0.051	0.732	0.739	0.745	0.741	1.01	1.02	1.01
		.6	0.047	0.046	0.049	0.041	0.716	0.724	0.731	0.704	1.01	1.02	0.98
		.8	0.047	0.043	0.045	0.027	0.689	0.706	0.712	0.67	1.02	1.03	0.97
MAR	(30%, 50%)	0	0.05	0.051	0.052	0.051	0.654	0.665	0.661	0.66	1.02	1.01	1.01
		.2	0.048	0.05	0.049	0.048	0.639	0.654	0.655	0.651	1.02	1.03	1.02
		.4	0.047	0.05	0.05	0.045	0.628	0.648	0.64	0.633	1.03	1.02	1.01
		.6	0.05	0.051	0.053	0.038	0.622	0.647	0.645	0.609	1.04	1.04	0.98
		.8	0.043	0.044	0.047	0.032	0.591	0.627	0.621	0.579	1.06	1.05	0.98

*Note*. Multivariate and univariate methods are compared for scenarios which vary in terms of missingness, percentage of missing data and degree of correlation between outcomes. The Holm method was applied to all scenarios to account for multiplicity. Monte Carlo standard errors for the simulation were consistent across methods. MCSE Range for FWER = (0.0016, 0.0023); MCSE Range for Power = (0.0034, 0.0049).

Abbreviations: LV, latent variable model; MM, multilevel multivariate model; UV, univariate model; MI+UV, multiple imputation followed by univariate model; ρ, correlation between outcomes.

## 6 DISCUSSION

In this paper, we have reviewed the statistical methodology that can be used to analyse multiple correlated outcomes in clinical trials. We have performed a simulation study to investigate differences in bias, FWER and disjunctive power achieved using the MM model, an LV model and a univariate model with (MI+UV) and without (UV) multiple imputation.

The simulation results suggest that the disjunctive power may be increased by using MM models as opposed to analysing each outcome separately with or without multiple imputation (UV and MI+UV). However, we found that the power gains were generally small unless the outcomes were strongly correlated or there were high levels of missing data. Pituch, Whittaker, and Chang (2016) and Snijders and Bosker (2012) reported efficiency gains for MM model compared to UV models in presence of missing data based on case studies.

When the pairwise correlations between the outcomes were weak, the power was reduced when using the MI+UV approach compared to using the UV approach. These findings are consistent with the results presented in Sullivan, White, Salter, Ryan, and Lee (2018), which state that MI may be less efficient than complete case analysis due to Monte Carlo simulation error. When missing values are MNAR, the MI+UV approach and the MM model produce very similar effect estimates and hence a similar level of bias. Both approaches provide some improvement over the UV model when the outcome correlations are .6 or higher. As expected, neither MI+UV nor MM removed the bias entirely. As a consequence, any inferences and conclusions made within the trial setting should be confirmed with sensitivity analyses under the alternative assumptions that the missing data are MNAR.

The MM model offers a computational advantage to the MI+UV approach as the MM model enables the analysis to be performed in just one step. In contrast, MI+UV method requires three steps: specifying the imputation model and performing the imputation; fitting the analysis model to each of the imputed data sets and combining the results across the imputed data sets.

When a single primary outcome is specified in a trial, the MM model can still be used for the analysis of secondary outcomes. Alternatively, when there are missing values in the primary outcome, both the primary and secondary outcomes may be analysed simultaneously using the MM model. In addition, the MM model allows for joint effects to be estimated although this should be documented in advance in a statistical analysis plan.

The results from the LV model are dependent on the constraints imposed on the model. In this paper, we fixed the latent factor variance. For a discussion of alternative constraints, see Skrondal and Rabe‐Hesketh (2004). A limitation of our simulation study is that we only considered normally distributed continuous outcomes. As further work, we suggest investigating continuous outcomes that follow alternative distributions, including skewed distributions or those with heavier tails.

The work focused on methods that have been previously suggested to analyse multiple outcomes in clinical trials. However, other methods are also available including copula models (Chen & Hanson, 2017; de Leon & Wu, 2011) and generalised estimation equations (GEE) (Prentice & Zhao, 1991; Teixeira‐Pinto & Normand, 2011). The GEE approach is robust to the misspecification of the correlation between the outcomes but has been shown to be less efficient estimates compared to the LV model (Teixeira‐Pinto & Normand, 2011).

## CONFLICT OF INTEREST

The authors have declared no conflict of interest.

### OPEN RESEARCH BADGES

This article has earned an Open Data badge for making publicly available the digitally‐shareable data necessary to reproduce the reported results. The data is available in the Supporting Information section.

This article has earned an open data badge “**Reproducible Research**” for making publicly available the code necessary to reproduce the reported results. The results reported in this article were reproduced partially due to their computational complexity.

## Supporting information



Appendix

Figures

Code & data
