# Supplementary material for: A comparison of methods for analysing multiple outcome measures in randomised controlled trials using a simulation study
Source: Biom J. 2020 Dec 14;63(3):599–615. doi: 10.1002/bimj.201900040 (PMC7984364; doi:10.1002/bimj.201900040)
Supplement: Supplementary file 1 — Appendix [file BIMJ-63-599-s001.docx]

**Appendix Simulation results**

**Table A.1a** FWER and disjunctive power when analysing *four continuous outcomes*

Multivariate and univariate methods are compared for scenarios which vary in terms of missingness, percentage of missing data and degree of correlation between outcomes. The Holm method was applied to all scenarios to account for multiplicity.

| **Type of**  **missingness** | **% of missing**  **values for each outcome** | $\rho$ | **Family wise error rate**  **(FWER)** | | | **Disjunctive power** | | | **Relative power**  **(vs. UV)** | |
| --- | --- | --- | --- | --- | --- | --- | --- | --- | --- | --- |
|  |  |  | UV | MI + UV | MM | UV | MI + UV | MM | MI+UV | MM |
| Complete | (0%, 0%,  0%, 0%) | 0 | 0.046 | - | 0.051 | 0.980 | - | 0.982 | - | 1.00 |
|  |  | 0.2 | 0.049 | - | 0.052 | 0.950 | - | 0.954 | - | 1.00 |
|  |  | 0.4 | 0.046 | - | 0.050 | 0.915 | - | 0.920 | - | 1.01 |
|  |  | 0.6 | 0.040 | - | 0.043 | 0.858 | - | 0.866 | - | 1.01 |
|  |  | 0.8 | 0.035 | - | 0.038 | 0.788 | - | 0.797 | - | 1.01 |
| MCAR | (15%, 25%,  15%, 25%) | 0 | 0.052 | 0.050 | 0.059 | 0.937 | 0.933 | 0.946 | 1.00 | 1.01 |
|  |  | 0.2 | 0.048 | 0.050 | 0.056 | 0.899 | 0.898 | 0.907 | 1.00 | 1.01 |
|  |  | 0.4 | 0.044 | 0.049 | 0.051 | 0.852 | 0.864 | 0.874 | 1.01 | 1.03 |
|  |  | 0.6 | 0.045 | 0.047 | 0.046 | 0.801 | 0.827 | 0.831 | 1.03 | 1.04 |
|  |  | 0.8 | 0.036 | 0.036 | 0.036 | 0.749 | 0.788 | 0.789 | 1.05 | 1.05 |
| MCAR | (20%, 30%,  40%, 50%) | 0 | 0.045 | 0.045 | 0.053 | 0.876 | 0.855 | 0.891 | 0.98 | 1.02 |
|  |  | 0.2 | 0.051 | 0.053 | 0.057 | 0.836 | 0.836 | 0.859 | 1.00 | 1.03 |
|  |  | 0.4 | 0.046 | 0.053 | 0.054 | 0.787 | 0.811 | 0.826 | 1.03 | 1.05 |
|  |  | 0.6 | 0.047 | 0.051 | 0.050 | 0.739 | 0.791 | 0.797 | 1.07 | 1.08 |
|  |  | 0.8 | 0.043 | 0.047 | 0.040 | 0.680 | 0.757 | 0.750 | 1.11 | 1.10 |
| MAR | (15%, 25%,  15%, 25%) | 0 | 0.052 | 0.050 | 0.058 | 0.938 | 0.931 | 0.945 | 0.99 | 1.01 |
|  |  | 0.2 | 0.048 | 0.049 | 0.053 | 0.902 | 0.901 | 0.913 | 1.00 | 1.01 |
|  |  | 0.4 | 0.048 | 0.050 | 0.053 | 0.849 | 0.865 | 0.874 | 1.02 | 1.03 |
|  |  | 0.6 | 0.043 | 0.047 | 0.049 | 0.802 | 0.829 | 0.834 | 1.03 | 1.04 |
|  |  | 0.8 | 0.039 | 0.039 | 0.039 | 0.748 | 0.785 | 0.784 | 1.05 | 1.05 |
| MAR | (20%, 30%,  40%, 50%) | 0 | 0.050 | 0.049 | 0.059 | 0.874 | 0.857 | 0.891 | 0.98 | 1.02 |
|  |  | 0.2 | 0.050 | 0.053 | 0.059 | 0.828 | 0.824 | 0.851 | 0.99 | 1.03 |
|  |  | 0.4 | 0.048 | 0.050 | 0.052 | 0.783 | 0.808 | 0.820 | 1.03 | 1.05 |
|  |  | 0.6 | 0.044 | 0.050 | 0.049 | 0.739 | 0.791 | 0.798 | 1.07 | 1.08 |
|  |  | 0.8 | 0.041 | 0.043 | 0.038 | 0.691 | 0.763 | 0.759 | 1.10 | 1.10 |

Key: MM = multilevel multivariate model; UV = univariate model; MI + UV = multiple imputation followed by univariate model; LV = Latent variable model; $\rho$ = correlation between outcomes. Note: Standard errors for the simulation were consistent across methods. SE Range for FWER = (0.0018, 0.0024); SE Range for Power = (0.0013, 0.0047)

**Table A.1b FWER and disjunctive power when analysing *two continuous and two binary outcomes (four ‘mixed’ outcomes)***  Multivariate and univariate methods are compared for scenarios which vary in terms of missingness, percentage of missing data and degree of correlation between outcomes. The Holm method was applied to all scenarios to account for multiplicity.

| **Type of**  **missingness** | **% of missing values for each outcome** | $\rho$ | **Family wise error rate**  **(FWER)** | | | **Disjunctive power** | | | | **Relative power**  **(vs. UV)** | |
| --- | --- | --- | --- | --- | --- | --- | --- | --- | --- | --- | --- |
|  |  |  | UV | MI + UV | MM | UV | MI + UV | MM | MI+UV | | MM |
| Complete | (0%, 0%,  0%, 0%) | 0 | 0.048 | - | 0.050 | 0.948 | - | 0.951 | - | | 1.00 |
|  |  | 0.2 | 0.044 | - | 0.047 | 0.908 | - | 0.912 | - | | 1.00 |
|  |  | 0.4 | 0.048 | - | 0.049 | 0.874 | - | 0.878 | - | | 1.01 |
|  |  | 0.6 | 0.040 | - | 0.041 | 0.821 | - | 0.827 | - | | 1.01 |
|  |  | 0.8 | 0.037 | - | 0.038 | 0.765 | - | 0.771 | - | | 1.01 |
| MCAR | (15%, 25%,  15%, 25%) | 0 | 0.050 | 0.046 | 0.052 | 0.883 | 0.863 | 0.891 | 0.98 | | 1.01 |
|  |  | 0.2 | 0.052 | 0.046 | 0.056 | 0.842 | 0.827 | 0.852 | 0.98 | | 1.01 |
|  |  | 0.4 | 0.047 | 0.044 | 0.050 | 0.803 | 0.801 | 0.821 | 1.00 | | 1.02 |
|  |  | 0.6 | 0.044 | 0.042 | 0.046 | 0.755 | 0.769 | 0.785 | 1.02 | | 1.04 |
|  |  | 0.8 | 0.044 | 0.040 | 0.045 | 0.706 | 0.731 | 0.749 | 1.04 | | 1.06 |
| MCAR | (20%, 30%,  40%, 50%) | 0 | 0.050 | 0.041 | 0.054 | 0.811 | 0.761 | 0.823 | 0.94 | | 1.01 |
|  |  | 0.2 | 0.049 | 0.041 | 0.052 | 0.774 | 0.744 | 0.796 | 0.96 | | 1.03 |
|  |  | 0.4 | 0.045 | 0.038 | 0.049 | 0.740 | 0.730 | 0.765 | 0.99 | | 1.03 |
|  |  | 0.6 | 0.046 | 0.039 | 0.052 | 0.703 | 0.715 | 0.746 | 1.02 | | 1.06 |
|  |  | 0.8 | 0.041 | 0.032 | 0.042 | 0.656 | 0.689 | 0.712 | 1.05 | | 1.09 |
| MAR | (15%, 25%,  15%, 25%) | 0 | 0.046 | 0.042 | 0.049 | 0.880 | 0.856 | 0.886 | 0.97 | | 1.01 |
|  |  | 0.2 | 0.051 | 0.047 | 0.054 | 0.841 | 0.829 | 0.852 | 0.99 | | 1.01 |
|  |  | 0.4 | 0.046 | 0.041 | 0.046 | 0.797 | 0.801 | 0.819 | 1.01 | | 1.03 |
|  |  | 0.6 | 0.046 | 0.044 | 0.051 | 0.757 | 0.773 | 0.786 | 1.02 | | 1.04 |
|  |  | 0.8 | 0.040 | 0.038 | 0.041 | 0.711 | 0.737 | 0.745 | 1.04 | | 1.05 |
| MAR | (20%, 30%,  40%, 50%) | 0 | 0.048 | 0.040 | 0.054 | 0.808 | 0.761 | 0.820 | 0.94 | | 1.02 |
|  |  | 0.2 | 0.051 | 0.043 | 0.055 | 0.760 | 0.728 | 0.783 | 0.96 | | 1.03 |
|  |  | 0.4 | 0.048 | 0.043 | 0.055 | 0.738 | 0.730 | 0.768 | 0.99 | | 1.04 |
|  |  | 0.6 | 0.043 | 0.035 | 0.045 | 0.688 | 0.703 | 0.731 | 1.02 | | 1.06 |
|  |  | 0.8 | 0.044 | 0.036 | 0.044 | 0.646 | 0.688 | 0.706 | 1.07 | | 1.09 |

\

Key: MM = multilevel multivariate model; UV = univariate model; MI + UV = multiple imputation followed by univariate model;; $\rho$ = correlation between outcomes. Note: Standard errors for the simulation were consistent across methods. SE Range for FWER = (0.0017, 0.0023); SE Range for Power = (0.0020, 0.0048)

**Table A.2a FWER and disjunctive power when analysing *two continuous outcomes with varying effect sizes***

Multivariate and univariate methods are compared for scenarios which vary in terms of missingness, percentage of missing data and degree of correlation between outcomes. The Holm method was applied to all scenarios to account for multiplicity.

| **Type of**  **missingness** | **% of missing values for each outcome** | $\rho$ | **Disjunctive Power** | | | **Relative power**  **(vs. UV)** | | |  |
| --- | --- | --- | --- | --- | --- | --- | --- | --- | --- |
|  |  |  | UV | MI + UV | MM | | MI+UV | MM | |
| Complete | (0%, 0%) | 0 | 0.775 | - | 0.789 | | - | 1.01 | |
|  |  | 0.2 | 0.754 | - | 0.763 | | - | 1.01 | |
|  |  | 0.4 | 0.738 | - | 0.747 | | - | 1.01 | |
|  |  | 0.6 | 0.729 | - | 0.738 | | - | 1.01 | |
|  |  | 0.8 | 0.717 | - | 0.726 | | - | 1.01 | |
| MCAR | (15%,25%) | 0 | 0.641 | 0.607 | 0.655 | | 0.95 | 1.02 | |
|  |  | 0.2 | 0.633 | 0.612 | 0.650 | | 0.97 | 1.03 | |
|  |  | 0.4 | 0.618 | 0.629 | 0.648 | | 1.02 | 1.05 | |
|  |  | 0.6 | 0.601 | 0.637 | 0.648 | | 1.06 | 1.08 | |
|  |  | 0.8 | 0.590 | 0.665 | 0.666 | | 1.13 | 1.13 | |
| MCAR | (30%,50%) | 0 | 0.475 | 0.374 | 0.499 | | 0.79 | 1.05 | |
|  |  | 0.2 | 0.476 | 0.394 | 0.508 | | 0.83 | 1.07 | |
|  |  | 0.4 | 0.453 | 0.435 | 0.500 | | 0.96 | 1.10 | |
|  |  | 0.6 | 0.442 | 0.497 | 0.512 | | 1.12 | 1.16 | |
|  |  | 0.8 | 0.443 | 0.560 | 0.551 | | 1.27 | 1.25 | |
| MAR | (15%,25%) | 0 | 0.649 | 0.612 | 0.665 | | 0.94 | 1.02 | |
|  |  | 0.2 | 0.630 | 0.611 | 0.650 | | 0.97 | 1.03 | |
|  |  | 0.4 | 0.616 | 0.624 | 0.644 | | 1.01 | 1.04 | |
|  |  | 0.6 | 0.601 | 0.638 | 0.645 | | 1.06 | 1.07 | |
|  |  | 0.8 | 0.592 | 0.665 | 0.668 | | 1.12 | 1.13 | |
| MAR | (30%,50%) | 0 | 0.455 | 0.367 | 0.480 | | 0.81 | 1.06 | |
|  |  | 0.2 | 0.461 | 0.383 | 0.490 | | 0.83 | 1.06 | |
|  |  | 0.4 | 0.444 | 0.419 | 0.490 | | 0.95 | 1.10 | |
|  |  | 0.6 | 0.430 | 0.471 | 0.496 | | 1.10 | 1.15 | |
|  |  | 0.8 | 0.427 | 0.553 | 0.544 | | 1.30 | 1.28 | |

Key: MM = multilevel multivariate model; UV = univariate model; MI + UV = multiple imputation followed by univariate model;$\rho$ = correlation between outcomes.

Note: Standard errors for the simulation were consistent across methods. The range of the simulation standard error (SE) was 0.003 to 0.005

**Table A.2b FWER and disjunctive power when analysing *four continuous outcomes with varying effect sizes***

Multivariate and univariate methods are compared for scenarios which vary in terms of missingness, percentage of missing data and degree of correlation between outcomes. The Holm method was applied to all scenarios to account for multiplicity.

| **Type of**  **missingness** | **% of missing values for**  **each outcome** | $\rho$ | **Disjunctive Power** | | | **Relative power**  **(vs. UV)** | |
| --- | --- | --- | --- | --- | --- | --- | --- |
|  |  |  | UV | MI + UV | MM | MI+UV | MM |
| Complete | (0%, 0%,  0%, 0%) | 0 | 0.799 | - | 0.812 | - | 1.02 |
|  |  | 0.2 | 0.743 | - | 0.757 | - | 1.02 |
|  |  | 0.4 | 0.717 | - | 0.732 | - | 1.02 |
|  |  | 0.6 | 0.676 | - | 0.689 | - | 1.02 |
|  |  | 0.8 | 0.635 | - | 0.649 | - | 1.02 |
| MCAR | (15%, 25%,  15%, 25%) | 0 | 0.652 | 0.646 | 0.683 | 0.99 | 1.05 |
|  |  | 0.2 | 0.616 | 0.630 | 0.646 | 1.02 | 1.05 |
|  |  | 0.4 | 0.600 | 0.620 | 0.644 | 1.03 | 1.07 |
|  |  | 0.6 | 0.558 | 0.618 | 0.626 | 1.11 | 1.12 |
|  |  | 0.8 | 0.531 | 0.613 | 0.619 | 1.15 | 1.17 |
| MCAR | (20%, 30%,  40%, 50%) | 0 | 0.510 | 0.486 | 0.552 | 0.95 | 1.08 |
|  |  | 0.2 | 0.476 | 0.483 | 0.532 | 1.02 | 1.12 |
|  |  | 0.4 | 0.442 | 0.498 | 0.517 | 1.13 | 1.17 |
|  |  | 0.6 | 0.423 | 0.534 | 0.538 | 1.26 | 1.27 |
|  |  | 0.8 | 0.386 | 0.572 | 0.553 | 1.48 | 1.43 |
| MAR | (15%, 25%,  15%, 25%) | 0 | 0.655 | 0.648 | 0.678 | 0.99 | 1.03 |
|  |  | 0.2 | 0.619 | 0.626 | 0.648 | 1.01 | 1.05 |
|  |  | 0.4 | 0.590 | 0.622 | 0.636 | 1.05 | 1.08 |
|  |  | 0.6 | 0.552 | 0.613 | 0.621 | 1.11 | 1.13 |
|  |  | 0.8 | 0.517 | 0.606 | 0.605 | 1.17 | 1.17 |
| MAR | (20%, 30%,  40%, 50%) | 0 | 0.484 | 0.479 | 0.528 | 0.99 | 1.09 |
|  |  | 0.2 | 0.459 | 0.479 | 0.508 | 1.04 | 1.11 |
|  |  | 0.4 | 0.437 | 0.500 | 0.510 | 1.15 | 1.17 |
|  |  | 0.6 | 0.415 | 0.513 | 0.519 | 1.24 | 1.25 |
|  |  | 0.8 | 0.376 | 0.555 | 0.543 | 1.48 | 1.44 |

Key: MM = multilevel multivariate model; UV = univariate model; MI + UV = multiple imputation followed by univariate model; LV = Latent variable model; $\rho$ = correlation between outcomes

**Figure A.1a Bias in estimating the intervention effects when simulating *two continuous outcomes* and data are MCAR or MAR.**

The blue dots represent the average of the estimated treatment effects ($\hat{\beta}$) for outcome 1. The red dots represent the average of the estimated treatment effects ($\hat{\beta}$) for outcome 2. The five dots (of either colour) clustered together represent different correlations between outcomes from 0 (left) to 0.8 (right) in increments of 0.2. Each graph corresponds to a different type and level of missing data as indicated. The true intervention effect is represented by the black horizontal line.

X Insert Figure A.1a X

**Figure A.1b Bias in estimating the intervention effects when simulating *two binary outcomes* and data are MCAR or MAR.**

The blue dots represent the average of the estimated treatment effects ($\hat{\beta}$) for outcome 1. The red dots represent the average of the estimated treatment effects ($\hat{\beta}$) for outcome 2. The five dots (of either colour) clustered together represents different correlations between outcomes from 0 (left) to 0.8 (right) in increments of 0.2. Each graph corresponds to different level and type of missing data as indicated.

X Insert Figure A.1b X

**Figure A.1c Bias in estimating the intervention effects when simulating *two mixed outcomes* and data are MCAR or MAR.**

The blue dots represent the average of the estimated treatment effects ($\hat{\beta}$) for outcome 1. The red dots represent the average of the estimated treatment effects ($\hat{\beta}$) for outcome 2. The five dots (of either colour) clustered together represent different correlations between outcomes from 0 (left) to 0.8 (right) in increments of 0.2. Each graph corresponds to a different level and type of missing data as indicated. The true intervention effect is represented by the black horizontal line.

X Insert Figure A.1c X

**Figure A.1d Bias in estimating the intervention effects when simulating two binary outcomes and data are MNAR**

The blue dots represent the average of the estimated treatment effects ($\hat{\beta}$) for outcome 1. The red dots represent the average of the estimated treatment effects ($\hat{\beta}$) for outcome 2. The five dots (of either colour) clustered together represent different correlations between outcomes from 0 (left) to 0.8 (right) in increments of 0.2. Each graph corresponds to a different level of missing data as indicated. The true intervention effect is represented by the black horizontal line.

X Insert Figure A.1d X

**Figure A.1e Bias in estimating the intervention effects when simulating one continuous and one binary (‘two mixed’) outcomes and data are MNAR**

The blue dots represent the average of the estimated treatment effect ($\hat{\beta}$) for outcome 1.The red dots represent the average of the estimated treatment effects ($\hat{\beta}$) for outcome 2. The five dots (of either colour) clustered together represent different correlations between outcomes from 0 (left) to 0.8 (right) in increments of 0.2. Each graph corresponds to a different level of missing data as indicated. The true intervention effect is represented by the black horizontal line.

X Insert Figure A.1e X

**Figure A.1f Bias in estimating the intervention effects when simulating four continuous outcomes and data are MNAR**

The four colours each represent the average of the estimated treatment effects for the different outcome. The five dots (of each colour) clustered together represent different correlations between the outcomes from 0 (left) to 0.8 (right) in increments of 0.2. Each graph corresponds to a different level of missing data as indicated. The true intervention effect is represented by the black horizontal line.

X Insert Figure A.1f X

**Figure A.1g Bias in estimating the intervention effects when simulating two continuous and two binary (‘four mixed’) outcomes and data are MNAR**

The four colours each represent the average of the estimated treatment effects for the different outcomes. The five dots (of each colour) clustered together represent different correlations between the outcomes from 0 (left) to 0.8 (right) in increments of 0.2. Each graph corresponds to a has different level of missing data as indicated. The true intervention effect is represented by the black horizontal line.

XX Insert Figure A.1g X

**Table A.3a Empirical standard error (EmpSE) of estimated intervention effect when analysing two continuous outcomes**

| **Type of**  **Missingness** ↓ | **% of missing values**  **for each outcome** ↓ | $\boldsymbol{\rho}$  ↓ | **EmpSE of estimated intervention effect on outcome 1** | | | | **EmpSE of estimated intervention effect on outcome 2** | | | |
| --- | --- | --- | --- | --- | --- | --- | --- | --- | --- | --- |
|  |  | **Method** → | UV | MI + UV | MM | LV | UV | MI + UV | MM | LV |
| Complete | (0%, 0%) | 0 | 0.123 | - | 0.123 | 0.123 | 0.124 | - | 0.124 | 0.124 |
|  |  | 0.2 | 0.123 | - | 0.123 | 0.123 | 0.124 | - | 0.124 | 0.124 |
|  |  | 0.4 | 0.125 | - | 0.125 | 0.125 | 0.124 | - | 0.124 | 0.124 |
|  |  | 0.6 | 0.124 | - | 0.124 | 0.124 | 0.123 | - | 0.123 | 0.123 |
|  |  | 0.8 | 0.125 | - | 0.125 | 0.128 | 0.124 | - | 0.124 | 0.128 |
| MCAR | (15%, 25%) | 0 | 0.134 | 0.137 | 0.134 | 0.134 | 0.145 | 0.153 | 0.145 | 0.145 |
|  |  | 0.2 | 0.134 | 0.135 | 0.134 | 0.134 | 0.143 | 0.145 | 0.142 | 0.142 |
|  |  | 0.4 | 0.135 | 0.134 | 0.134 | 0.134 | 0.143 | 0.142 | 0.141 | 0.141 |
|  |  | 0.6 | 0.136 | 0.134 | 0.133 | 0.133 | 0.145 | 0.141 | 0.140 | 0.140 |
|  |  | 0.8 | 0.133 | 0.130 | 0.129 | 0.131 | 0.143 | 0.135 | 0.134 | 0.137 |
| MCAR | (30%, 50%) | 0 | 0.148 | 0.156 | 0.148 | 0.148 | 0.177 | 0.197 | 0.177 | 0.177 |
|  |  | 0.2 | 0.148 | 0.153 | 0.148 | 0.148 | 0.176 | 0.187 | 0.175 | 0.175 |
|  |  | 0.4 | 0.148 | 0.148 | 0.147 | 0.147 | 0.177 | 0.175 | 0.171 | 0.171 |
|  |  | 0.6 | 0.149 | 0.147 | 0.146 | 0.146 | 0.175 | 0.168 | 0.165 | 0.165 |
|  |  | 0.8 | 0.147 | 0.143 | 0.141 | 0.142 | 0.175 | 0.157 | 0.154 | 0.155 |
| MAR | (15%, 25%) | 0 | 0.133 | 0.136 | 0.133 | 0.133 | 0.145 | 0.153 | 0.145 | 0.145 |
|  |  | 0.2 | 0.134 | 0.135 | 0.134 | 0.134 | 0.143 | 0.146 | 0.143 | 0.143 |
|  |  | 0.4 | 0.136 | 0.136 | 0.135 | 0.135 | 0.143 | 0.142 | 0.141 | 0.141 |
|  |  | 0.6 | 0.134 | 0.132 | 0.132 | 0.132 | 0.143 | 0.139 | 0.138 | 0.138 |
|  |  | 0.8 | 0.135 | 0.132 | 0.132 | 0.133 | 0.144 | 0.136 | 0.135 | 0.137 |
| MAR | (30%, 50%) | 0 | 0.147 | 0.155 | 0.147 | 0.147 | 0.178 | 0.199 | 0.178 | 0.178 |
|  |  | 0.2 | 0.148 | 0.153 | 0.148 | 0.148 | 0.181 | 0.193 | 0.180 | 0.180 |
|  |  | 0.4 | 0.148 | 0.149 | 0.146 | 0.146 | 0.180 | 0.179 | 0.175 | 0.175 |
|  |  | 0.6 | 0.150 | 0.150 | 0.148 | 0.148 | 0.180 | 0.173 | 0.169 | 0.169 |
|  |  | 0.8 | 0.148 | 0.143 | 0.142 | 0.143 | 0.180 | 0.160 | 0.157 | 0.158 |

Key: MM = multivariate multilevel model; UV = univariate model; MI + UV = multiple imputation followed by univariate model; LV = Latent variable model; $\rho*$ = correlation between outcomes.

**Table A.3b Empirical standard error (EmpSE) of estimated intervention effect when analysing two binary outcomes**

| **Type of**  **missingness**↓ | **% of missing values for each outcome** ↓ | $\boldsymbol{\rho}$ **↓** | **EmpSE of estimated intervention effect on outcome 1** | | | **EmpSE of estimated intervention effect on outcome 2** | | |
| --- | --- | --- | --- | --- | --- | --- | --- | --- |
|  |  | **Method** → | UV | MI + UV | MM | UV | MI + UV | MM |
| Complete | (0%, 0%) | 0 | 0.138 | - | 0.141 | 0.137 | - | 0.137 |
|  |  | 0.2 | 0.138 | - | 0.141 | 0.140 | - | 0.140 |
|  |  | 0.4 | 0.137 | - | 0.140 | 0.139 | - | 0.139 |
|  |  | 0.6 | 0.138 | - | 0.141 | 0.139 | - | 0.139 |
|  |  | 0.8 | 0.139 | - | 0.142 | 0.138 | - | 0.139 |
| MCAR | (15%, 25%) | 0 | 0.150 | 0.150 | 0.151 | 0.162 | 0.161 | 0.162 |
|  |  | 0.2 | 0.150 | 0.150 | 0.151 | 0.160 | 0.159 | 0.160 |
|  |  | 0.4 | 0.150 | 0.149 | 0.151 | 0.159 | 0.158 | 0.158 |
|  |  | 0.6 | 0.150 | 0.149 | 0.150 | 0.160 | 0.157 | 0.157 |
|  |  | 0.8 | 0.149 | 0.147 | 0.148 | 0.161 | 0.153 | 0.155 |
| MCAR | (30%, 50%) | 0 | 0.166 | 0.165 | 0.167 | 0.198 | 0.196 | 0.199 |
|  |  | 0.2 | 0.166 | 0.165 | 0.167 | 0.197 | 0.194 | 0.203 |
|  |  | 0.4 | 0.166 | 0.164 | 0.166 | 0.195 | 0.190 | 0.194 |
|  |  | 0.6 | 0.167 | 0.165 | 0.167 | 0.199 | 0.190 | 0.193 |
|  |  | 0.8 | 0.165 | 0.161 | 0.163 | 0.199 | 0.184 | 0.188 |
| MAR | (15%, 25%) | 0 | 0.149 | 0.149 | 0.150 | 0.159 | 0.158 | 0.159 |
|  |  | 0.2 | 0.151 | 0.151 | 0.152 | 0.159 | 0.159 | 0.160 |
|  |  | 0.4 | 0.151 | 0.150 | 0.152 | 0.160 | 0.158 | 0.158 |
|  |  | 0.6 | 0.149 | 0.148 | 0.149 | 0.162 | 0.159 | 0.160 |
|  |  | 0.8 | 0.151 | 0.148 | 0.150 | 0.160 | 0.154 | 0.156 |
| MAR | (30%, 50%) | 0 | 0.165 | 0.164 | 0.166 | 0.200 | 0.197 | 0.201 |
|  |  | 0.2 | 0.167 | 0.166 | 0.172 | 0.201 | 0.197 | 0.201 |
|  |  | 0.4 | 0.167 | 0.166 | 0.167 | 0.202 | 0.196 | 0.199 |
|  |  | 0.6 | 0.166 | 0.163 | 0.166 | 0.202 | 0.192 | 0.196 |
|  |  | 0.8 | 0.168 | 0.163 | 0.170 | 0.204 | 0.186 | 0.191 |

Key: MM = multivariate multilevel model; UV = univariate model; MI + UV = multiple imputation followed by univariate model; $\rho$ = correlation between outcomes.

**Table A.3c Empirical standard error (EmpSE) of estimated intervention effect when analysing ‘mixed’ outcomes (one continuous and one binary)**

| **Type of**  **missingness**↓ | **% of missing values for each outcome**↓ | $\boldsymbol{\rho}$ **↓** | **EmpSE of estimated intervention effect on outcome 1** | | | | **EmpSE of estimated intervention effect on outcome 2** | | | |
| --- | --- | --- | --- | --- | --- | --- | --- | --- | --- | --- |
|  |  | **Method** → | UV | MI + UV | MM | LV | UV | MI + UV | MM | LV |
| Complete | (0%, 0%) | 0 | 0.124 | - | 0.160 | 0.160 | 0.124 | - | 0.160 | 0.161 |
|  |  | 0.2 | 0.124 | - | 0.158 | 0.158 | 0.124 | - | 0.158 | 0.165 |
|  |  | 0.4 | 0.125 | - | 0.158 | 0.158 | 0.125 | - | 0.158 | 0.186 |
|  |  | 0.6 | 0.122 | - | 0.159 | 0.159 | 0.122 | - | 0.159 | 0.272 |
|  |  | 0.8 | 0.124 | - | 0.160 | 0.160 | 0.124 | - | 0.160 | 0.504 |
| MCAR | (15%,25%) | 0 | 0.134 | 0.134 | 0.134 | 0.134 | 0.185 | 0.184 | 0.185 | 0.186 |
|  |  | 0.2 | 0.134 | 0.135 | 0.134 | 0.134 | 0.182 | 0.181 | 0.182 | 0.190 |
|  |  | 0.4 | 0.135 | 0.135 | 0.134 | 0.134 | 0.184 | 0.181 | 0.182 | 0.218 |
|  |  | 0.6 | 0.134 | 0.133 | 0.133 | 0.133 | 0.185 | 0.179 | 0.180 | 0.325 |
|  |  | 0.8 | 0.136 | 0.134 | 0.134 | 0.134 | 0.181 | 0.172 | 0.174 | 0.687 |
| MCAR | (30%,50%) | 0 | 0.150 | 0.150 | 0.150 | 0.150 | 0.224 | 0.221 | 0.225 | 0.228 |
|  |  | 0.2 | 0.148 | 0.149 | 0.148 | 0.148 | 0.226 | 0.221 | 0.226 | 0.237 |
|  |  | 0.4 | 0.148 | 0.148 | 0.147 | 0.147 | 0.226 | 0.218 | 0.222 | 0.275 |
|  |  | 0.6 | 0.148 | 0.146 | 0.146 | 0.146 | 0.225 | 0.211 | 0.216 | 0.389 |
|  |  | 0.8 | 0.149 | 0.145 | 0.145 | 0.146 | 0.228 | 0.203 | 0.210 | 0.553 |
| MAR | (15%,25%) | 0 | 0.133 | 0.133 | 0.133 | 0.133 | 0.184 | 0.183 | 0.184 | 0.185 |
|  |  | 0.2 | 0.135 | 0.135 | 0.135 | 0.135 | 0.183 | 0.182 | 0.183 | 0.190 |
|  |  | 0.4 | 0.135 | 0.135 | 0.134 | 0.134 | 0.183 | 0.181 | 0.182 | 0.219 |
|  |  | 0.6 | 0.133 | 0.132 | 0.132 | 0.132 | 0.182 | 0.176 | 0.177 | 0.313 |
|  |  | 0.8 | 0.134 | 0.131 | 0.131 | 0.132 | 0.183 | 0.174 | 0.176 | 0.515 |
| MAR | (30%,50%) | 0 | 0.149 | 0.150 | 0.150 | 0.150 | 0.229 | 0.225 | 0.230 | 0.233 |
|  |  | 0.2 | 0.150 | 0.151 | 0.150 | 0.150 | 0.231 | 0.225 | 0.230 | 0.243 |
|  |  | 0.4 | 0.149 | 0.149 | 0.148 | 0.148 | 0.230 | 0.220 | 0.226 | 0.278 |
|  |  | 0.6 | 0.148 | 0.146 | 0.146 | 0.146 | 0.232 | 0.216 | 0.222 | 0.389 |
|  |  | 0.8 | 0.149 | 0.145 | 0.145 | 0.146 | 0.229 | 0.205 | 0.212 | 0.566 |

Key: MM = multivariate multilevel model; UV = univariate model; MI + UV = multiple imputation followed by univariate model; LV = Latent variable model; $\rho$ = correlation between outcomes.
