# Supplementary material for: A comparison of methods for analysing multiple outcome measures in randomised controlled trials using a simulation study
Source: Biom J. 2020 Dec 14;63(3):599–615. doi: 10.1002/bimj.201900040 (PMC7984364; doi:10.1002/bimj.201900040)
Supplement: Supplementary file 3 — Code & data [file BIMJ-63-599-s003.zip › Code_and_data/READ_ME.pdf]

A comparison of methods for analysing multiple outcome measures in randomised controlled trials using a simulation study

Victoria Vickerstaff, Gareth Ambler and Rumana Omar

November 2020

Code: Victoria Vickerstaff

Email: v.vickerstaff@ucl.ac.uk

1. Introduction

This document explains the necessary steps to reproduce the tables displayed in the paper *'A comparison of methods for analysing multiple outcome measures in randomised controlled trials using a simulation study'*.

The paper has two main sections:

1) Simulation study
2) Case study.

The data used to produce the results for both sections are provided and code is provided to reproduce the tables. Additionally, the code to re-run the simulation studies are provided to ensure reproducibility. Details regarding both the simulation study and case study sections are provided below.

1.1. Simulation study

We performed a simulation study to evaluate the bias in the estimates of the intervention effects and the power of detecting true intervention effects observed when using selected methods. Different simulation scenarios were constructed by varying the number of outcomes, the type of outcomes, the degree of correlations between the outcomes and the proportions and mechanisms of missing data.

We used simulations to compare the multivariate multilevel (MM) and latent variable (LV) models to univariate models with (MI+UV) and without multiple imputation (UV) with respect to power and FWER.

The multivariate multilevel (MM) was performed using MLwiN via R. The univariate model with and without multiple imputation were performed in R. The latent variable model was performed in Stata.

The simulation results used to produce Tables 3a, 3b and 3c have been provided. These are saved in the folder "*Table3_Figure1_datasets*".

1.2. Case studies

Two case studies are provided in the paper: 1) LiGHT and 2) Ten Top Tips. Both datasets are confidential and the main study data. Consequently, the provided data here has been masked and

thus they are different from the real data used in the paper. The case study data files have been saved in the folder "*case_study_data*". The results produced from these datasets are similar, but not exactly the same, as those provided in the paper.

2. Requirements

MLwiN, R and Stata and must be installed in order to perform the simulation studies and to re-create the results from the case studies. The following requirements were used for this paper.

2.1. Requirements for MLwiN.

MLWiN version 3.04

2.2. Requirements for R.

R version 3.6.1 (2019-07-05)

Platform: i386-w64-mingw32/i386 (32-bit)

Running under: Windows >= 8 x64 (build 9200)

Attached base packages:

stats4 stats graphics grDevices utils datasets methods base

Other attached packages:

mice_3.6.0 R2MLwiN_0.8-6 coda_0.19-3 memisc_0.99.17.2 lattice_0.20-38 multcomp_1.4-10 TH.data_1.0-10 MASS_7.3-51.4 survival_2.44-1.1 bindata_0.9-19 nlme_3.1-140 lme4_1.1-21 Matrix_1.2-17 mvtnorm_1.0-11 plyr_1.8.4 ggplot2_3.2.0 haven_2.1.1 dplyr_0.8.3

2.3. Requirements for Stata.

Stata version 14.2

Running under: Windows- 64-bit

Packages attached:

- Gllamm
- runmlwin

3. Files

The list of files and folders in the Code_and_Data.zip file:

README.pdf

case_study_data

intermediate_results

Table3_Figure1_datasets

saved

1_Reproduce_MM_and_MI_results_in_paper.R

user_made_functions.R

2_Reproduce_LM_results_in_paper.do

lv_analyses.do

lv_results.do

3_Case_studies.do

The folders "*intermediate_results*" and "*saved*" are intentionally empty. These are used as part of the simulation study to store datasets and intermediate results.

4. How to run R code to produce results in Table 3a, 3b and 3c and Figure 1: Simulation study

The data files used to create Tables 3a-3c and Figure 1 are saved in the folder "*Table3_Figure1_datasets*". Within this folder there are 4 files: 2cts_presults.dta, 2bin_presults.dta, 2mix_presults.dta and 2cts_mnar_coef.dta. These are used to reproduce Tables 3a, 3b, 3c and Figure 1 respectively.

To reproduce these tables exactly, the R script "1_Table3a_3c_3c_figure1.R" needs to be run. The file paths need to be changed on lines 25 and 26.

Once the file paths have been amended, Table 3a is reproduced by running lines 33-35.

The output from R is:

```
> table3a$power
   type_missingness percent_missing corr FWER-UV FWER-MI+UV FWER-MM FWER-LV Power-UV Power-MI+UV Power-MM Power-LV Relativepower-MI+UV Relativepower-MM Relativepower-LV
1              none        (0%, 0%)  0.0   0.052        NaN   0.054   0.054    0.922         NaN    0.926    0.926                 NaN             1.00             1.00
2              none        (0%, 0%)  0.2   0.048        NaN   0.051   0.051    0.894         NaN    0.900    0.900                 NaN             1.01             1.01
3              none        (0%, 0%)  0.4   0.052        NaN   0.054   0.054    0.873         NaN    0.877    0.877                 NaN             1.00             1.00
4              none        (0%, 0%)  0.6   0.045        NaN   0.048   0.048    0.838         NaN    0.844    0.844                 NaN             1.01             1.01
5              none        (0%, 0%)  0.8   0.038        NaN   0.040   0.048    0.798         NaN    0.806    0.814                 NaN             1.01             1.02
6              mcar      (15%, 25%)  0.0   0.048      0.044   0.051   0.050    0.852       0.811    0.860    0.859                0.95             1.01             1.01
7              mcar      (15%, 25%)  0.2   0.049      0.045   0.054   0.053    0.825       0.804    0.835    0.835                0.97             1.01             1.01
8              mcar      (15%, 25%)  0.4   0.048      0.051   0.053   0.053    0.803       0.807    0.818    0.818                1.00             1.02             1.02
9              mcar      (15%, 25%)  0.6   0.048      0.051   0.050   0.049    0.769       0.790    0.797    0.797                1.03             1.04             1.04
10             mcar      (15%, 25%)  0.8   0.044      0.046   0.044   0.052    0.741       0.779    0.779    0.787                1.05             1.05             1.06
11             mcar      (30%, 50%)  0.0   0.050      0.028   0.055   0.055    0.721       0.532    0.738    0.736                0.74             1.02             1.02
12             mcar      (30%, 50%)  0.2   0.050      0.029   0.058   0.057    0.704       0.568    0.726    0.725                0.81             1.03             1.03
13             mcar      (30%, 50%)  0.4   0.051      0.044   0.056   0.055    0.689       0.648    0.718    0.716                0.94             1.04             1.04
14             mcar      (30%, 50%)  0.6   0.049      0.055   0.054   0.055    0.667       0.694    0.711    0.711                1.04             1.07             1.07
15             mcar      (30%, 50%)  0.8   0.044      0.052   0.045   0.053    0.643       0.708    0.704    0.709                1.10             1.09             1.10
16              mar      (15%, 25%)  0.0   0.052      0.048   0.055   0.055    0.849       0.808    0.858    0.858                0.95             1.01             1.01
17              mar      (15%, 25%)  0.2   0.049      0.045   0.053   0.052    0.825       0.805    0.834    0.834                0.98             1.01             1.01
18              mar      (15%, 25%)  0.4   0.050      0.051   0.053   0.053    0.794       0.800    0.809    0.809                1.01             1.02             1.02
19              mar      (15%, 25%)  0.6   0.047      0.051   0.052   0.052    0.765       0.784    0.788    0.788                1.02             1.03             1.03
20              mar      (15%, 25%)  0.8   0.044      0.046   0.046   0.054    0.726       0.761    0.761    0.769                1.05             1.05             1.06
21              mar      (30%, 50%)  0.0   0.050      0.032   0.056   0.056    0.713       0.539    0.729    0.728                0.76             1.02             1.02
22              mar      (30%, 50%)  0.2   0.048      0.032   0.052   0.052    0.696       0.567    0.718    0.716                0.81             1.03             1.03
23              mar      (30%, 50%)  0.4   0.048      0.042   0.053   0.052    0.678       0.631    0.702    0.701                0.93             1.04             1.03
24              mar      (30%, 50%)  0.6   0.050      0.054   0.053   0.053    0.656       0.670    0.696    0.696                1.02             1.06             1.06
25              mar      (30%, 50%)  0.8   0.040      0.052   0.047   0.055    0.627       0.678    0.677    0.685                1.08             1.08             1.09
```

If you wish to look at the corresponding MCSEs which are discussed in the table footer you can type "*table3a$se*"

Table 3b is reproduced by running lines 38-40. The output from R is:

```
>     table3b$power
```

	type_missingness	percent_missing	corr	FWER-UV	FWER-MI+UV	FWER-MM	FWER-LV	Power-UV	Power-MI+UV	Power-MM	Power-LV	Relativepower-MI+UV	Relativepower-MM	Relativepower-LV
1	none	(0%, 0%)	0.0	0.053	NaN	0.064	0.053	0.919	NaN	0.920	0.919	NaN	1.00	1.00
2	none	(0%, 0%)	0.2	0.047	NaN	0.052	0.047	0.902	NaN	0.903	0.902	NaN	1.00	1.00
3	none	(0%, 0%)	0.4	0.049	NaN	0.058	0.049	0.893	NaN	0.895	0.893	NaN	1.00	1.00
4	none	(0%, 0%)	0.6	0.053	NaN	0.064	0.052	0.863	NaN	0.865	0.861	NaN	1.00	1.00
5	none	(0%, 0%)	0.8	0.047	NaN	0.058	0.036	0.840	NaN	0.843	0.821	NaN	1.00	0.98
6	mcar	(15%, 25%)	0.0	0.051	0.049	0.070	0.050	0.848	0.838	0.847	0.844	0.99	1.00	1.00
7	mcar	(15%, 25%)	0.2	0.048	0.044	0.063	0.044	0.836	0.824	0.838	0.828	0.99	1.00	0.99
8	mcar	(15%, 25%)	0.4	0.044	0.043	0.061	0.042	0.816	0.811	0.824	0.810	0.99	1.01	0.99
9	mcar	(15%, 25%)	0.6	0.050	0.049	0.068	0.046	0.795	0.792	0.805	0.786	1.00	1.01	0.99
10	mcar	(15%, 25%)	0.8	0.046	0.043	0.063	0.036	0.768	0.776	0.792	0.750	1.01	1.03	0.98
11	mcar	(30%, 50%)	0.0	0.051	0.045	0.066	0.048	0.729	0.704	0.734	0.721	0.97	1.01	0.99
12	mcar	(30%, 50%)	0.2	0.048	0.042	0.058	0.043	0.715	0.693	0.720	0.698	0.97	1.01	0.98
13	mcar	(30%, 50%)	0.4	0.047	0.043	0.062	0.042	0.701	0.681	0.710	0.678	0.97	1.01	0.97
14	mcar	(30%, 50%)	0.6	0.052	0.046	0.063	0.041	0.687	0.671	0.707	0.656	0.98	1.03	0.95
15	mcar	(30%, 50%)	0.8	0.048	0.041	0.063	0.034	0.667	0.672	0.708	0.627	1.01	1.06	0.94
16	mar	(15%, 25%)	0.0	0.049	0.046	0.068	0.048	0.849	0.840	0.850	0.846	0.99	1.00	1.00
17	mar	(15%, 25%)	0.2	0.050	0.046	0.067	0.048	0.832	0.824	0.835	0.827	0.99	1.00	0.99
18	mar	(15%, 25%)	0.4	0.046	0.042	0.060	0.042	0.812	0.806	0.819	0.808	0.99	1.01	1.00
19	mar	(15%, 25%)	0.6	0.048	0.045	0.063	0.044	0.792	0.789	0.801	0.783	1.00	1.01	0.99
20	mar	(15%, 25%)	0.8	0.049	0.044	0.064	0.036	0.767	0.769	0.786	0.745	1.00	1.02	0.97
21	mar	(30%, 50%)	0.0	0.052	0.048	0.069	0.050	0.722	0.700	0.726	0.715	0.97	1.01	0.99
22	mar	(30%, 50%)	0.2	0.049	0.043	0.058	0.044	0.707	0.686	0.709	0.691	0.97	1.00	0.98
23	mar	(30%, 50%)	0.4	0.048	0.042	0.064	0.042	0.686	0.673	0.697	0.670	0.98	1.02	0.98
24	mar	(30%, 50%)	0.6	0.051	0.044	0.065	0.041	0.672	0.665	0.689	0.649	0.99	1.03	0.97
25	mar	(30%, 50%)	0.8	0.048	0.041	0.065	0.034	0.657	0.661	0.687	0.616	1.01	1.05	0.94

If you wish to look at the corresponding MCSEs which are discussed in the table footer you can type "*table3b$se*"

Table 3b is reproduced by running lines 43-45. The output from R is:

```
>     table3c$power
```

	type_missingness	percent_missing	corr	FWER-UV	FWER-MI+UV	FWER-MM	FWER-LV	Power-UV	Power-MI+UV	Power-MM	Power-LV	Relativepower-MI+UV	Relativepower-MM	Relativepower-LV
1	none	(0%, 0%)	0.0	0.052	NaN	0.054	0.054	0.861	NaN	0.864	0.864	NaN	1.00	1.00
2	none	(0%, 0%)	0.2	0.053	NaN	0.054	0.054	0.836	NaN	0.840	0.840	NaN	1.00	1.00
3	none	(0%, 0%)	0.4	0.051	NaN	0.052	0.052	0.815	NaN	0.819	0.816	NaN	1.00	1.00
4	none	(0%, 0%)	0.6	0.046	NaN	0.048	0.040	0.794	NaN	0.798	0.779	NaN	1.01	0.98
5	none	(0%, 0%)	0.8	0.045	NaN	0.046	0.027	0.765	NaN	0.770	0.723	NaN	1.01	0.95
6	mcar	(15%, 25%)	0.0	0.050	0.049	0.051	0.051	0.777	0.776	0.784	0.784	1.00	1.01	1.01
7	mcar	(15%, 25%)	0.2	0.050	0.049	0.052	0.051	0.756	0.757	0.765	0.763	1.00	1.01	1.01
8	mcar	(15%, 25%)	0.4	0.048	0.048	0.050	0.049	0.740	0.746	0.750	0.746	1.01	1.01	1.01
9	mcar	(15%, 25%)	0.6	0.050	0.047	0.050	0.039	0.720	0.734	0.738	0.710	1.02	1.03	0.99
10	mcar	(15%, 25%)	0.8	0.047	0.045	0.047	0.029	0.698	0.715	0.722	0.680	1.02	1.03	0.97
11	mcar	(30%, 50%)	0.0	0.049	0.050	0.051	0.051	0.655	0.668	0.665	0.663	1.02	1.02	1.01
12	mcar	(30%, 50%)	0.2	0.048	0.050	0.050	0.049	0.648	0.658	0.660	0.656	1.02	1.02	1.01
13	mcar	(30%, 50%)	0.4	0.049	0.054	0.053	0.048	0.632	0.653	0.651	0.640	1.03	1.03	1.01
14	mcar	(30%, 50%)	0.6	0.050	0.052	0.053	0.040	0.623	0.656	0.653	0.612	1.05	1.05	0.98
15	mcar	(30%, 50%)	0.8	0.046	0.046	0.050	0.029	0.601	0.638	0.638	0.589	1.06	1.06	0.98
16	mar	(15%, 25%)	0.0	0.050	0.049	0.051	0.051	0.782	0.779	0.786	0.786	1.00	1.01	1.01
17	mar	(15%, 25%)	0.2	0.048	0.047	0.051	0.051	0.757	0.755	0.764	0.763	1.00	1.01	1.01
18	mar	(15%, 25%)	0.4	0.052	0.049	0.052	0.051	0.732	0.739	0.745	0.741	1.01	1.02	1.01
19	mar	(15%, 25%)	0.6	0.047	0.046	0.049	0.041	0.716	0.724	0.731	0.704	1.01	1.02	0.98
20	mar	(15%, 25%)	0.8	0.047	0.043	0.045	0.027	0.689	0.706	0.712	0.670	1.02	1.03	0.97
21	mar	(30%, 50%)	0.0	0.050	0.051	0.052	0.051	0.654	0.665	0.661	0.660	1.02	1.01	1.01
22	mar	(30%, 50%)	0.2	0.048	0.050	0.049	0.048	0.639	0.654	0.655	0.651	1.02	1.03	1.02
23	mar	(30%, 50%)	0.4	0.047	0.050	0.050	0.045	0.628	0.648	0.640	0.633	1.03	1.02	1.01
24	mar	(30%, 50%)	0.6	0.050	0.051	0.053	0.038	0.622	0.647	0.645	0.609	1.04	1.04	0.98
25	mar	(30%, 50%)	0.8	0.043	0.044	0.047	0.032	0.591	0.627	0.621	0.579	1.06	1.05	0.98

If you wish to look at the corresponding MCSEs which are discussed in the table footer you can type "*table3c$se*"

Figure 1 is produced by combining four separate plots. Initially lines 48-64 need to be run. Then the four separate plots are produced.

Line 66 produces the plot for low missingness (0%, 15%) as follows:

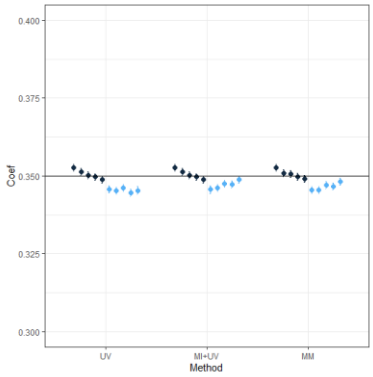

Line 67 produces the plot for medium missingness (0%, 30%) as follows:

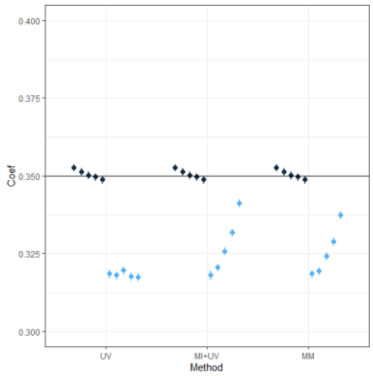

Line 68 produces the plot for high missingness (0%, 30%) as follows:

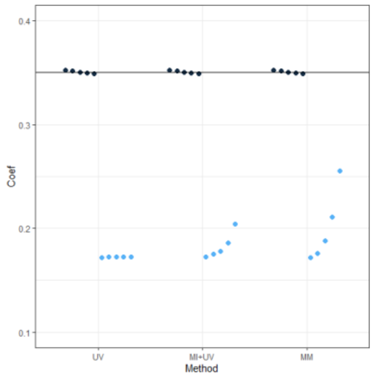

Line 69 produces the plot for overlapping missingness (30%, 50%) as follows:

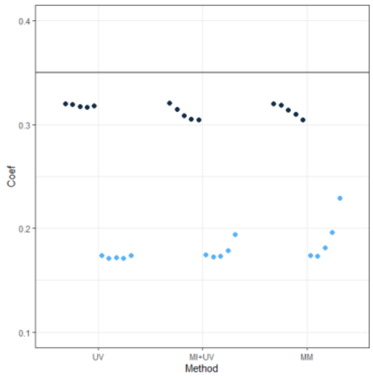

5. How to reproduce the simulation study

The simulation study can be reproduced using the files "*2_Reproduce_MM_and_MI_results_in_paper.R*" and "*3_Reproduce_LM_reuslts_in_paper.do*". To run the R file MLwiN will need to be installed on the computer.

Two file paths will need to be amended to run the coding: 1) where the Code_and_data folder is unzipped and saved 2) program file to MLwiN. In order to run MLwiN via R, the absolute file path to the folder where the program files are stored needs to be provided (e.g. C:/Program Files/MLwiN v3.04/)

In "*2_Reproduce_MM_and_MI_results_in_paper.R*" the paths can be amended on lines 35-36. In "*3_Reproduce_LM_reuslts_in_paper.do*" the file path can be changed on line 27.

These files call the "*user_made_functions.R*", "*lv_analyses.do*" and "*lv_results.do*" files.

Each scenario is run separately. The code is written to produce 100 simulations, however, this can be amended by changing '*nsims'* as desired. Datasets are generated as part of the R file – "*2_Reproduce_MM_and_MI_results_in_paper.R*" and are stored in the folder "*saved*". It is necessary to generate the datasets using the R code before running the stata .do file. Intermediate results are saved every 10 simulations, these are saved in the folder "*intermediate_results*".

6. How to run Stata code to produce results in Tables 4 and Table 5: Case studies

Because the LiGHT and Ten Top Tips datasets are confidential, the data has been masked and thus they are different from the real data used in the paper. The case study data files have been saved in the folder "*case_study_data*".

After changing the directory where the unzipped files are located, you can load the data and calculate the results table running the commands provided in "*3_Case_studies.do*". The results obtained are provided below. These are similar (but not identical) to Table 4 and Table 5.

The paths to be changed on lines 21 and 22. In stata, the MLwiN path needs to include the 'mlwin.exe' file, e.g. "C:\Program Files\MLwiN v3.04\mlwin.exe"

To obtain the output for the LiGHT Case study (table 4), run the code lines 21 to 272. The following output is obtained from running these lines. The output is similar to that provided in Table 4:

	method	n	mean_diff	se	lower_ci	upper_ci	pvalue
1	UV-EQ5D	652	-0.002	0.008	-0.018	0.013	0.768
2	UV-GQL	586	-0.460	0.457	-1.356	0.436	0.315
3	UV-GUI	602	0.012	0.008	-0.003	0.027	0.115
4	MI+UV-EQ5D	651	-0.003	0.008	-0.018	0.013	0.754
5	MI+UV-GQL	651	-0.529	0.450	-1.412	0.353	0.240
6	MI+UV-GUI	651	0.013	0.008	-0.002	0.028	0.099
7	MM-EQ5D	652	-0.002	0.008	-0.018	0.013	0.765
8	MM-GQL	652	-0.418	0.455	-1.310	0.474	0.358
9	MM-GUI	652	0.012	0.008	-0.003	0.027	0.124
10	LV-EQ5D	652	-0.003	0.008	-0.018	0.013	0.711
11	LV-GQL	652	-0.382	0.440	-1.244	0.481	0.386
12	LV-GUI	652	0.011	0.007	-0.003	0.025	0.130

To obtain the output for the Ten Top Tips case study (table 5), run the code lines 21-25 and then 275 to 521. The following output is obtained from running lines. The output is similar to that provided in Table 5:

	method	n	mean_diff	se	lower_ci	upper_ci	pvalue
1	UV-WEIGHT	383	-0.052	0.018	-0.088	-0.016	0.005
2	UV-WAIST	378	-0.069	0.048	-0.164	0.026	0.156
3	UV-GLUCOSE	330	-0.278	0.318	-0.901	0.345	0.382
4	MI+UV-WEIGHT	383	-0.052	0.018	-0.088	-0.016	0.005
5	MI+UV-WAIST	383	-0.072	0.048	-0.167	0.022	0.136
6	MI+UV-GLUCOSE	383	-0.042	0.040	-0.120	0.036	0.289
7	MM-WEIGHT	388	-0.053	0.018	-0.089	-0.017	0.004
8	MM-WAIST	388	-0.071	0.048	-0.166	0.023	0.138
9	MM-GLUCOSE	388	-0.314	0.315	-0.931	0.303	0.319
10	LV-WEIGHT	388	-0.059	0.019	-0.096	-0.023	0.002
11	LV-WAIST	388	-0.075	0.046	-0.166	0.015	0.257
12	LV-GLUCOSE	388	-0.206	0.181	-0.561	0.150	0.103
